# Suppression of inflammatory arthritis by the parasitic worm product ES-62 is associated with epigenetic changes in synovial fibroblasts

**DOI:** 10.1371/journal.ppat.1010069

**Published:** 2021-11-08

**Authors:** Marlene Corbet, Miguel A. Pineda, Kun Yang, Anuradha Tarafdar, Sarah McGrath, Rinako Nakagawa, Felicity E. Lumb, Colin J. Suckling, William Harnett, Margaret M. Harnett

**Affiliations:** 1 Institute of Infection, Immunity and Inflammation, University of Glasgow, Glasgow, United Kingdom; 2 Immunity and Cancer, Francis Crick Institute, London, United Kingdom; 3 Strathclyde Institute of Pharmacy and Biomedical Sciences, University of Strathclyde, Glasgow, United Kingdom; 4 Department of Pure and Applied Chemistry, University of Strathclyde, Glasgow, United Kingdom; James Cook University, AUSTRALIA

## Abstract

ES-62 is the major secreted protein of the parasitic filarial nematode, *Acanthocheilonema viteae*. The molecule exists as a large tetramer (MW, ~240kD), which possesses immunomodulatory properties by virtue of multiple phosphorylcholine (PC) moieties attached to *N*-type glycans. By suppressing inflammatory immune responses, ES-62 can prevent disease development in certain mouse models of allergic and autoimmune conditions, including joint pathology in collagen-induced arthritis (CIA), a model of rheumatoid arthritis (RA). Such protection is associated with functional suppression of “pathogenic” hyper-responsive synovial fibroblasts (SFs), which exhibit an aggressive inflammatory and bone-damaging phenotype induced by their epigenetic rewiring in response to the inflammatory microenvironment of the arthritic joint. Critically, exposure to ES-62 *in vivo* induces a stably-imprinted CIA-SF phenotype that exhibits functional responses more typical of healthy, Naïve-SFs. Consistent with this, ES-62 “rewiring” of SFs away from the hyper-responsive phenotype is associated with suppression of ERK activation, STAT3 activation and miR-155 upregulation, signals widely associated with SF pathogenesis. Surprisingly however, DNA methylome analysis of Naïve-, CIA- and ES-62-CIA-SF cohorts reveals that rather than simply preventing pathogenic rewiring of SFs, ES-62 induces further changes in DNA methylation under the inflammatory conditions pertaining in the inflamed joint, including targeting genes associated with ciliogenesis, to programme a novel “resolving” CIA-SF phenotype. In addition to introducing a previously unsuspected aspect of ES-62’s mechanism of action, such unique behaviour signposts the potential for developing DNA methylation signatures predictive of pathogenesis and its resolution and hence, candidate mechanisms by which novel therapeutic interventions could prevent SFs from perpetuating joint inflammation and destruction in RA. Pertinent to these translational aspects of ES-62-behavior, small molecule analogues (SMAs) based on ES-62’s active PC-moieties mimic the rewiring of SFs as well as the protection against joint disease in CIA afforded by the parasitic worm product.

## Author summary

The Hygiene Hypothesis proposes that the recent eradication of parasitic worms and other pathogens has resulted in chronically unbalanced, hyperactive immune systems that likely contribute to the corresponding dramatically increased incidence of allergic, autoimmune and metabolic conditions worldwide. Collectively, epidemiological and experimental animal model data supporting this hypothesis have focused interest in developing “helminth therapies”, based on infections with worms or their immunomodulatory products. Reflecting this, ES-62, an anti-inflammatory protein we discovered in the secretions of the filarial nematode *Acanthocheilonema viteae*, can prevent disease initiation and progression in mouse models of asthma, dermatitis, RA, SLE and the comorbidities arising from obesity-accelerated ageing. We now show that its protection against arthritis is associated with an ability to stably reprogram stromal cells in the joint away from a pathogenic bone-damaging population to a novel “safe” phenotype. Signposting of such pathogenic and “safe” molecular signatures may now provide starting points for developing novel therapies to prevent joint disease in RA. In the broader context, our discovery that a defined parasitic worm product can reprogram host cell responses furthers both our understanding of the host-pathogen interaction and identifies new directions for developing anthelmintics and therapies to resolve inflammation and induce tissue repair in humans.

## Introduction

Rheumatoid Arthritis (RA) is a chronic autoimmune inflammatory disease that targets articular joints, transforming the synovium into an inflamed, hyperplastic and invasive pannus that results ultimately in cartilage and bone destruction [1]. Such joint destruction in RA and in mouse models of inflammatory arthritis (e.g., collagen-induced arthritis [CIA]), has long been associated with the chronic autoimmune inflammation that results from dysregulated T helper cell responses [2]. Nevertheless, more recently, interest has also focused on the important contributions that synovial fibroblasts (SFs) make to pathogenesis, from the early onset of the disease through to established inflammation and joint destruction [1]. During pathogenesis, SFs adopt a mesenchymal/fibrotic phenotype [3], undergoing epigenetic rewiring in response to the hypoxia and inflammation generated in the microenvironment of the arthritic joint [4,5]. This rewiring is evidenced by changes in their global DNA methylation status, particularly the hypomethylation of promoter regions associated with the de-repression of inflammatory genes driving SF transformation [1,4,6–8]. Collectively, expression of these inflammatory genes (e.g., cytokines and matrix metalloproteinases [MMPs]) drives cellular infiltration, pannus formation and, via the impact of RANKL on osteoclastogenesis, the cartilage and bone damage leading to joint destruction [1,4,5,7]. Indeed, recent studies have now provided direct evidence that such hyper-responsive SFs drive both joint inflammation and bone damage in mouse models of RA [9]. ES-62-mediated protection (and that of small molecule analogues [SMAs] based on its active PC-moiety) against CIA is associated with its ability to resolve the autoimmune IL-17-driven responses that would otherwise promote the pathogenic microenvironment of the arthritic joint: ES-62 achieves this by targeting TLR4 to downregulate aberrant MyD88 signalling and reset homeostatic immunoregulation, primarily by suppressing CD4^+^ and γδ T cell-derived IL-17 production and restoring levels of regulatory B cells (Bregs) [10–13]. However, ES-62 also acts to suppress the hyper-inflammatory phenotype of CIA-SFs [12] and prevent osteoclastogenesis [14].

Our aim, therefore, was to understand the mechanisms by which SFs become rewired to the hyper-responsive phenotype that drives joint pathology in the CIA mouse model and how ES-62 subverts these processes to elicit a functionally hypo-responsive CIA-SF phenotype. Specifically, we investigated the impact of the local pro-inflammatory environment pertaining in CIA on both the acute signalling and stable epigenetic mechanisms driving induction of the pathogenic hyper-responsive SF phenotype. Reflecting their high levels of expression in the arthritic joint, we now show that chronic exposure to IL-1β and IL-17 *in vitro* can recapitulate SF reprogramming, specifically the global DNA hypomethylation resulting from downregulation of DNA methyltransferase-1 (DNMT1), and that both the acute (cytokine and MMP production) and chronic (remodelling to a stable hyper-responsive phenotype) SF responses to these pathogenic cytokines are dependent on ERK and STAT3 signalling. ES-62 acts to rewire SFs away from the hyper-responsive phenotype during CIA, and this is associated with suppression of such cytokine-driven ERK and STAT3 activation. Critically however, ES-62 does not simply maintain the naïve, non-arthritic DNA methylation status but rather induces a distinct and stably-imprinted “resolving” CIA-SF phenotype. This unique protection provides a first step towards potentially identifying DNA methylation signatures predictive of pathogenesis and its resolution and hence, candidate mechanisms by which novel therapeutic interventions could prevent SFs from perpetuating inflammation and bone destruction in the joint.

## Results

### Exposure to ES-62 *in vivo* induces a stably modulated CIA-SF phenotype

**Fig 1:**
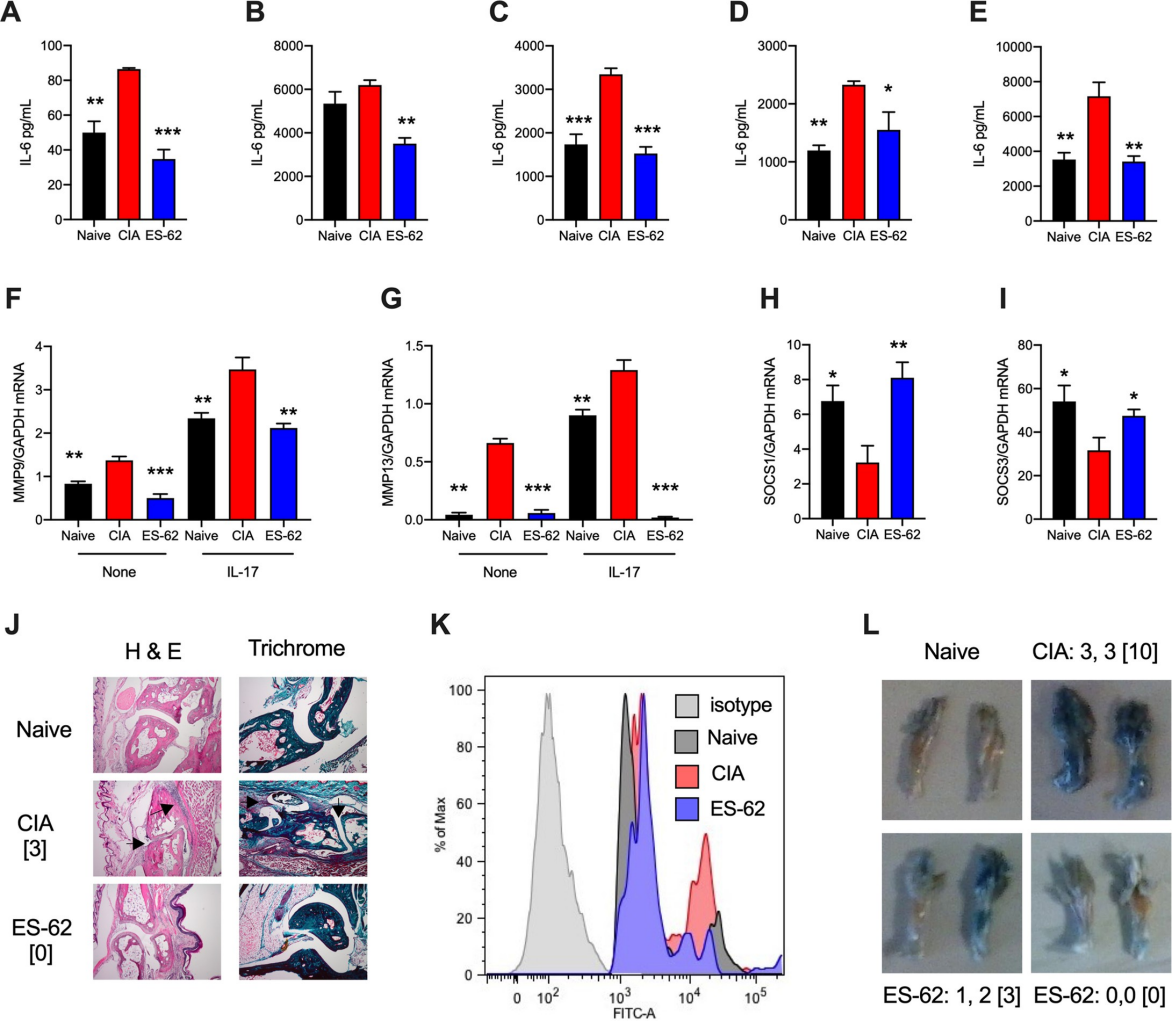
CIA induces a “hyper-responsive” SF phenotype that is counteracted by ES-62. SFs from Naïve, CIA and ES-62-CIA (ES-62) mice were incubated overnight in (**A**) medium or medium containing (**B**) IL-17, (**C**) IL-1β, (**D**) LPS or (**E**) BLP, and IL-6 release measured. Data are mean (of means of triplicates) values ± SEM of n = 3 cultures representative of 3–7 cultures. The pooled data revealed that CIA-SFs showed 1.486 ± 0.180-fold (n = 7, p<0.05) and ES-62-SFs, 0.873 ± 0.099-fold (n = 4) basal IL-6 release relative to Naïve-SFs and IL-17-stimulated CIA-SFs exhibited x2.29 ± 0.57-fold (n = 3, p<0.05) release compared to Naïve-IL-17 controls, whilst treatment with ES-62 reduced this hyper-production (x 1.87 ± 0.62-fold, n = 3). SFs were analysed for mRNA levels of MMP9 and MMP13 (**F and G**) or for SOCS1 and SOCS3 (**H and I**), following incubation overnight in medium (None) or where indicated, medium containing IL-17. Data are from single experiments representative of at least two and presented as mean (of means of triplicates) values ± SEM of n = 3 cultures, assayed by qRT-PCR. (**J**) Joint pathology showing representative sections from mice, with articular scores of the joint examined [indicated]. Arrows show the cartilage erosion and cellular infiltration in CIA sections. (**K**) Flow cytometric analysis of the hypoxic status of joint SFs determined by pimonidazole *in vivo*. (**L)** Representative images of joint vascular leakage (Evan’s Blue), where articular score for individual paws and [total mouse score] are from one experiment representative of two independent models. Throughout, SFs were pooled from individual mice to generate representative explant cohorts with articular scores: (**A-E, and I**) CIA, 3.17 ± 1.38, n = 6; ES-62, 0.5 ± 0.22, n = 6; (**F-H)** CIA, 3.66 ± 1.5, n = 6; ES-62, 1.5 ± 1.15, n = 6; (**K**) CIA, 4.667 ±1.99, n = 6; ES-62, 0.8 ± 0.8, n = 5. In all panels, *p<0.05; **p<0.01; ***p<0.001 relative to CIA SFs.

SFs from ES-62-treated CIA mice exhibit reduced spontaneous and IL-17-stimulated IL-6 release *ex vivo*, relative to those from control CIA mice [12]. To investigate whether this reflects stable rewiring of their functional phenotype, SF explant cultures (passaged for 3–4 weeks) from non-arthritic (Naïve; no CIA induction) and PBS- (CIA; disease control) and ES-62-treated mice undergoing CIA (ES-62) were examined for release of proinflammatory cytokines, chemokines and MMPs (**Fig 1**). We now show that both the increased spontaneous release of IL-6 in CIA- relative to Naïve-SFs and the ES-62 rescue of CIA-SF responses back towards the naïve functional phenotype is evident even after such sustained culture of SFs, *ex vivo* (**Fig 1A**). Moreover, IL-17-, IL-1β-, LPS- and BLP-stimulated release of IL-6 by SFs from PBS-treated CIA mice was similarly suppressed by *in vivo* ES-62 treatment (**Fig 1B–1E**), as was the spontaneous and IL-17- and LPS-stimulated CCL2 release by SFs from CIA mice (**S1A and S1B Fig**). This pattern of suppressed cytokine and chemokine release was found to be mirrored at the mRNA level (**S1C and S1D Fig**), analysis that also revealed spontaneous and IL-17-stimulated induction of MMP9 and MMP13 to be similarly downregulated in ES-62-CIA-, relative to CIA-, SFs (**Fig 1F and 1G**). At the same time, whilst the key negative regulators of such proinflammatory signalling, SOCS1 and SOCS3 [15, 16] are downregulated in CIA-SFs, exposure to ES-62 rescues their expression towards the higher levels observed in Naïve-SFs (**Fig 1H and 1I**). Reflecting this observed differential imprinting of pro-inflammatory and cartilage/extracellular matrix-degrading SF activities, whilst the joint damage observed in CIA-mice (**Fig 1J**) is associated with increased levels of hypoxia in joint cells (**Fig 1K**) and induction of vascular leakage (**Fig 1L**), these pathologies are countered by ES-62 (**Fig 1J–1L**).

### DNA hypomethylation associated with the CIA-SF phenotype can be mimicked by exposure of Naïve-SFs to IL-17 and IL-1β *in vitro*

**Fig 2:**
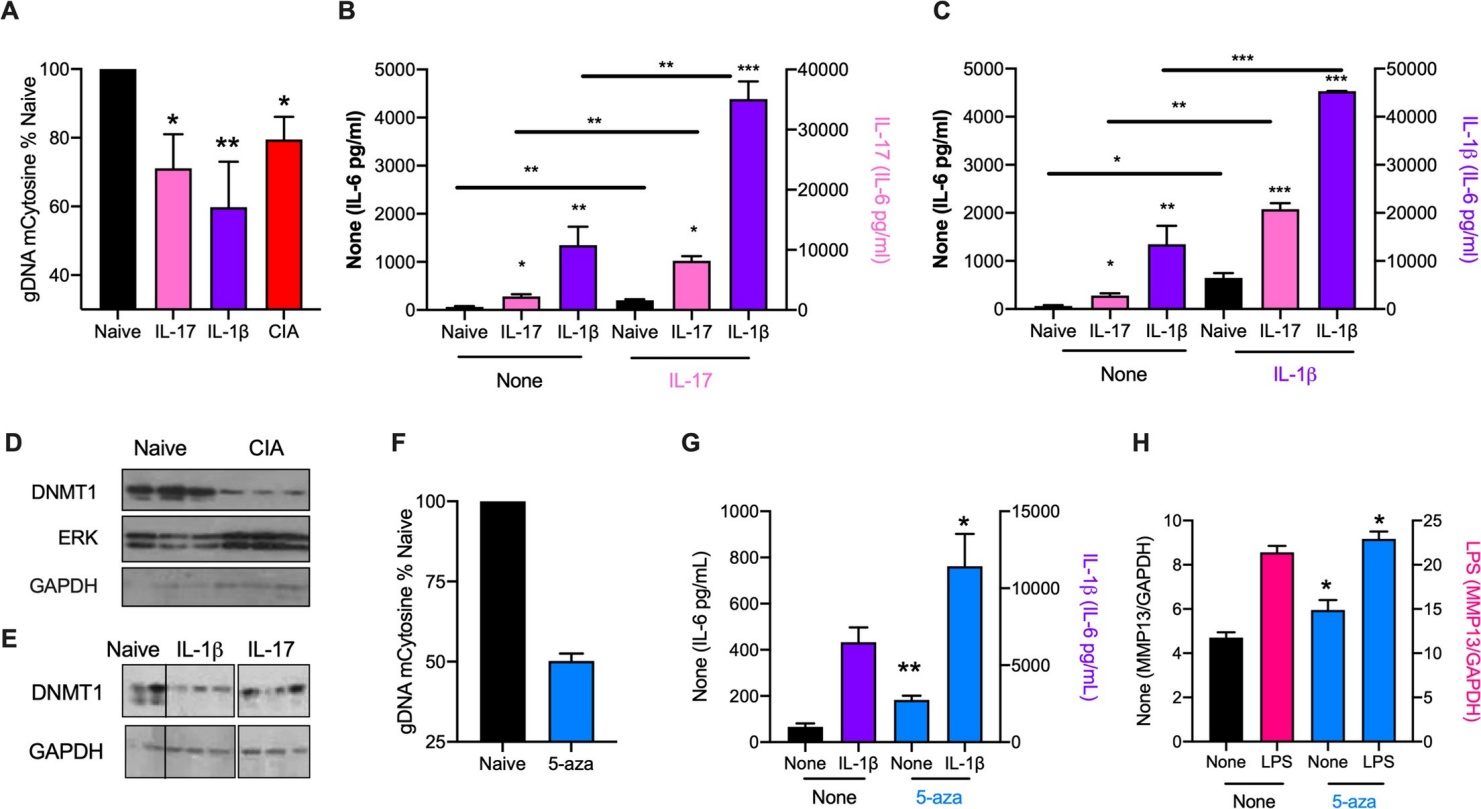
IL-1β and IL-17 rewire naïve SFs to a CIA-like phenotype. **(A)** Global methylation status of Naïve-SFs incubated in medium alone (Naïve) or chronically treated with IL-17 or IL-1β, compared to that of CIA-SFs. Data (means ± SEM) are from 3–5 independent experiments, normalized to “Naïve” controls. *p<0.05 and **p<0.01 are relative to Naïve-SFs. The CIA-SFs were from 3 independent models (articular scores: 3.6 ± 1.5, n = 6; 4.5 ± 1.3, n = 10 and 2.5 ± 0.8, n = 12). (**B and C)** Naïve-SFs incubated in medium alone (Naïve) or chronically treated with IL-17 or IL-1β were re-challenged with medium (None; **B and C**), or medium containing IL-17 (**B**) or IL-1β (**C**) and levels of IL-6 release determined. Data are means ± SEM of n = 3 independent cultures, where *p<0.05, **p<0.01 or ***p<0.001 are relative to the relevant Naïve-SF group (same control “None” data for **B and C**). Data in **B** are representative of 2 independent experiments. (**D**) Western blot analysis of DNMT1 expression in Naïve- and CIA- (articular score 3.6 ± 1.5, n = 6) SFs: each lane represents independent cultures and data (means ± SEM) were quantitated (DNMT1/GAPDH ratios normalized to Naïve controls) by ImageJ as Naïve, 1 ± 0.262 and CIA, 0.063 ± 0.004, **p<0.01. **(E)** Western blot analysis of DNMT1 expression in Naïve-SFs chronically treated with medium alone (Naïve) or containing IL-1β or IL-17, where lanes represent individual cultures. The data were quantitated (DNMT1/GAPDH ratios normalized to Naïve controls) by ImageJ as Naïve, 1 ± 0.22; IL-1β, 0.12 ± 0.02 and IL-17, 0.40 ± 0.14. **(F-H)** Naïve-SFs were chronically incubated in medium alone (None) or containing 5-aza and then assessed for **(F)** global DNA methylation status, **(G)** IL-6 release in response to IL-1β challenge and **(H)** MMP13 mRNA levels in response to LPS-stimulation. Data are presented as means ± SEM, where n = 3 independent cultures (**F** and **G**) or means ± SD, n = 3 triplicate analyses (**H**) and *p<0.05 or **p<0.01 relative to the appropriate sample lacking 5-aza (“None”).

Mirroring the DNA hypomethylation accompanying transformation of SFs to a pathogenic hyper-responsive phenotype in RA, CIA-SFs exhibit hypomethylated global DNA relative to those from naïve mice: such rewiring can be mimicked by sustained *in vitro* treatment of Naïve-SFs with either IL-17 or IL-1β (**Fig 2A**), cytokines that act on SFs to synergistically drive joint inflammation and damage [17, 18] and are suppressed by ES-62 *in vivo* [10]. The functional relevance of this epigenetic rewiring of SFs resulting from chronic exposure to these cytokines (particularly IL-1β) is illustrated by their accompanying hyper-responsiveness, in terms of spontaneous and IL-17- or IL-1β-stimulated IL-6 hyper-production (**Fig 2B and 2C**), that is reminiscent of that seen in CIA-, relative to Naïve-, SFs. Global DNA hypomethylation is associated with reduced expression of DNA methyltranferase-1 (DNMT1), and consistent with this, we found reduced expression of this enzyme in CIA-SFs (**Fig 2D**) and IL-1β- and, to a lesser extent, IL-17-treated Naïve-SFs (**Fig 2E**), compared to their control Naïve cohorts. Moreover, exposure to the DNMT1 inhibitor, 5-azacytidine (5-aza) reduces global DNA methylation in naïve SFs and again, this is associated with increased spontaneous and stimulated IL-6 secretion and MMP13 expression (**Fig 2F–2H**), providing corroborative evidence for this epigenetic mechanism of cytokine-induced SF hyper-responsiveness in CIA.

### IL-17- and IL-1β-induction of SF rewiring is associated with chronic ERK and STAT3 signalling

**Fig 3:**
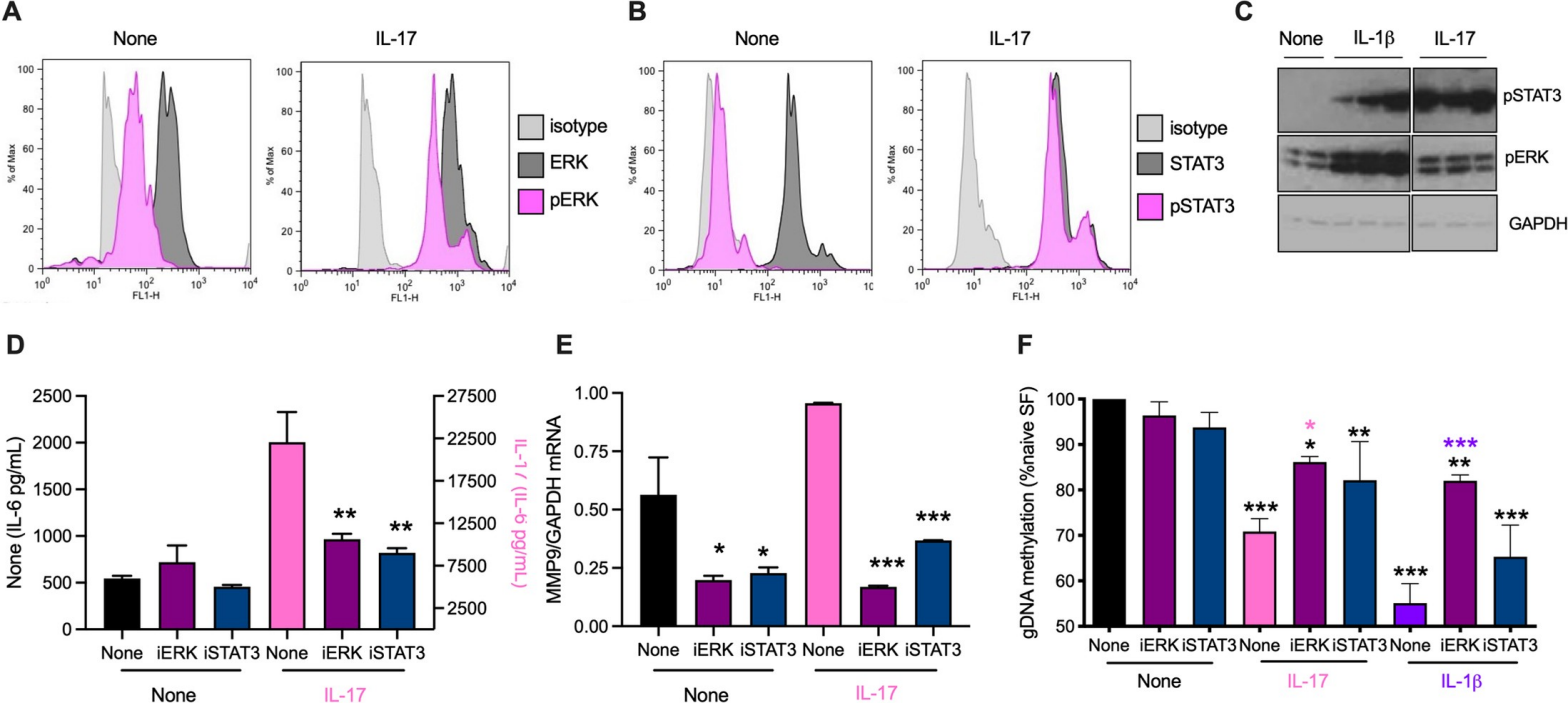
IL-17 and IL-1β stimulate ERK and STAT3 in SFs. Flow cytometric analysis of (**A**) ERK and (**B**) STAT3 activation, determining the relative expression of dually phosphorylated versus total ERK or phosphorylated STAT3 versus total STAT3 expression, following incubation of Naïve-SFs in medium alone or containing IL-17 for 20 min. (**C**) Naïve-SFs chronically incubated in medium alone (None) or containing IL-1β or IL-17 were analysed for pSTAT3, pERK and GAPDH expression by Western blot: individual lanes represent independent SF cultures. The data were quantitated (pSTAT3 or pERK/GAPDH ratios normalized to “None” controls) by ImageJ (Mean ± range, n = 2 for Naïve-SFs and Mean ± SEM, n = 3 for CIA- and ES-62-CIA SFs). For pSTAT3: None, 1 ± 0.51; IL-1β, 11.53 ± 5.06 and IL-17, 22.09 ± 5.14 and pERK: None, 1 ± 0.24; IL-1β, 2.65 ± 0.29 and IL-17, 1.14 ± 0.13. Naïve-SFs, pretreated with iERK or iSTAT3 for two hours, were incubated in medium alone (None) or with IL-17 overnight and levels of IL-6 release (**D**) or MMP9 mRNA (**E**) measured. (**F**) Naïve-SFs pretreated with iERK and iSTAT3 for two hours were chronically incubated in medium alone (None) or containing IL-17 or IL-1β and then their global DNA methylation assessed. Data are mean values ± SEM, n = 3 independent cultures and where *p<0.05, **p<0.01 and ***p<0.001 relative to appropriate None control (**D and E**) and None/None control (**F**) and where for iERK, *p <0.05 (pink) is relative to its IL-17 control and ***p<0.001 (purple) is relative to its IL-1β control (**F**).

IL-17 signals acutely via ERK MAPkinase and STAT3 in Naïve-SFs (**Fig 3A and 3B**). Extending previous studies implicating roles for these pathways in cytokine driven RA pathogenesis [18–22], we now show sustained activation of ERK (predominantly ERK2) and particularly, STAT3 following chronic stimulation with IL-17 or IL-1β (**Fig 3C**). Moreover, the MEK inhibitor PD98059 (iERK) and STAT3 inhibitor 5.15DPP (iSTAT3) not only suppress acute IL-17-stimulated IL-6, MMP9 and MMP13 expression, but also prevent chronic IL-17- and IL-1β-mediated global DNA hypomethylation (**Fig 3D–3F and S1E–S1G Fig**), indicating that such sustained signalling contributes to induction of the stably hyper-responsive SF phenotype (**Fig 2**). Further support is provided by our findings that CIA-SFs, appear to exhibit stronger and/or more prolonged ERK and STAT3 activation than Naïve-SFs in response to IL-17 and that exposure of CIA-mice to ES-62 suppresses this ERK and STAT3 (the latter back towards basal levels at 10 mins post-stimulation) hyper-responsiveness (**S1H and S1I Fig**). Whether such downmodulation of the hyper-responsive CIA-SF phenotype *in vivo* reflects direct or indirect effects of ES-62 was considered: whilst *in vitro* exposure to ES-62 does not impact on basal IL-6 production by Naïve-SFs, it does inhibit both the elevated basal release by CIA-SFs as well as the IL-17-stimulated production of this cytokine by Naïve-SFs (**S2A Fig**). Likewise, FACE analysis shows that *in vitro* exposure to ES-62 similarly suppresses the IL-17-stimulated, but not basal, ERK and STAT3 activation of Naïve SFs and this inhibition of IL-17-stimulated ERK activation in both Naïve- and CIA SFs has been corroborated by flow cytometry (**S2B–S2I Fig**). Thus, these data support the idea that ES-62 may have a direct effect on SFs *in vivo*.

### ES-62 targets microRNA networks in CIA-SFs

**Fig 4:**
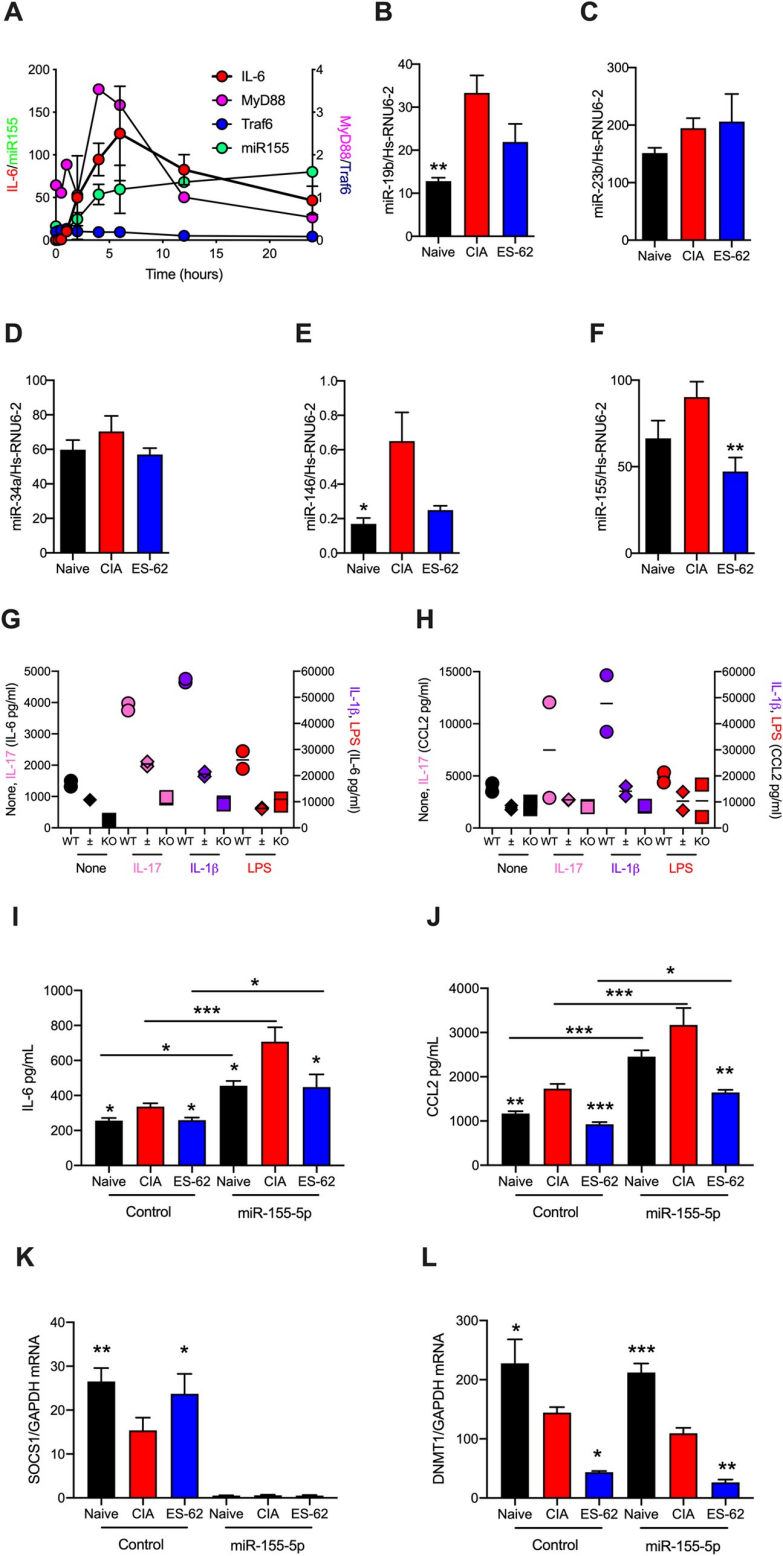
miR-146 and -155 are potential targets of ES-62 in suppressing the hyper-responsive CIA-SF phenotype. (**A**) Naïve-SFs were stimulated with LPS for the indicated times and miR-155, MyD88, Traf6 and IL-6 mRNA levels measured. Data are presented as mean values ± SEM, n = 4 for miR-155, mean ± range, n = 2 for IL-6 and single experiments for the other mediators. (**B-F**) Expression levels of the indicated miRs were determined for SFs from Naïve, CIA (articular score: 4.67 ± 2, n = 6) and ES-62-CIA (0.8 ± 0.8, n = 6) mice stimulated with LPS for 6 h and data presented as mean values ± SEM, n = 4–6 independent cultures and where *p<0.05 and **p< 0.01 relative to the CIA group. Although miR-19b or- 146 expression in ES-62-CIA-SFs was not significantly reduced (p = 0.06 and 0.07, respectively) when compared to that of CIA-SFs, it was not significantly elevated relative to that in the Naïve-SF controls. **(G and H)** SFs from wild type and miR-155 heterozygous (±) and homozygous (KO) C57BL/6 mice were incubated with the indicated stimuli and IL-6 **(G)** and CCL2 **(H)** release measured. Data shown represent the mean values (of triplicates) for two mice of each phenotype. **(I-L)** SFs from Naïve, CIA- (articular score 3.17 ± 1.38, n = 6) and ES-62-CIA- (articular score 0.5 ± 0.22, n = 6) mice were transfected for 24 hours with miR-155-5p mimic (50 nM) or control miR and then IL-6 and CCL2 release **(I and J)** and SOCS1 and DNMT1 mRNA levels **(L and L)** assessed. Data are from a single experiment and are expressed as the mean values (of triplicates) ± SEM, n = three independent cultures and where *p<0.05, **p<0.01, *** p<0.001. The IL-6 and CCL2 data were validated in an independent set of three cultures and further DNMT1 mRNA data are shown in Fig 5.

MicroRNAs (miRs) have been implicated in bridging JAK/STAT and MAPkinase signalling with epigenetic rewiring and consequent transcriptional reprogramming, with miR-146 and miR-155 proposed to be major species associated with SF pathogenesis in RA: indeed, both are elevated in fibroblast-like synoviocytes from RA patients (RA SFs) and have been associated with disease activity/severity [23–27]. Of a range of miRs implicated in regulating IL-1R/TLR and cytokine signalling and/or exhibiting modulated expression levels in CIA and human RA, we found miRs-19b, -23b, -34a, -146 and -155 to be expressed in SFs from Naïve and CIA mice. Moreover, and perhaps consistent with the proposed proinflammatory roles of TLRs in CIA/RA pathogenesis [28, 29], LPS/TLR4 signalling substantially increases miR-155 levels in Naïve-SFs and this increase is associated with similar kinetics of MyD88 and IL-6 (although not Traf6) upregulation, at the mRNA level (**Fig 4A**). We therefore investigated whether the TLR4/MyD88-dependent protective effects of ES-62 against pathogenic IL-1β/TLR- and cytokine-signalling and consequent joint damage were associated with modulation of expression of our panel of SF-associated miRs in CIA-SFs back towards the levels found in Naïve-SFs: this revealed that ES-62 indeed acted to reduce the elevated levels of miR-19b, -146 and -155 pertaining in CIA-SFs (**Fig 4B–4F**).

Interestingly, however, although miR-155 has been associated with driving pro-inflammatory innate and adaptive immune responses in RA and CIA [30–32], its role(s) in SF pathogenesis remains unclear [23–26,33]. Indeed, early studies reported that miR-155 plays a joint-protective role in RA-SFs, having been associated with downregulation of MMP1 and MMP3, mediators that promote SF proliferation and invasion, as well as bone destruction [23–26,33]. However, these studies also reported that miR-155 did not impact on MMP9 or MMP13 expression or modulate spontaneous or stimulated (TLR ligands/cytokines) TGFβ or IL-6 production [27,34]. Moreover, miR-155 was recently shown to drive (spontaneous and TNFα-stimulated) pathogenic responses (proliferation as well as IL-1β and IL-6 production) via downregulation of its target FOXO3 [35–37], which acts to suppress these functional outcomes in RA-SFs [38]. Thus, given the interactions of miR-155 with multiple TLR and cytokine (IL-1β, IL-10, IL-17, TNFα, GM-CSF) pathways implicated in regulating arthritogenesis [25,26], we further investigated the role of this element in SF pathogenesis and ES-62-mediated protection.

Firstly, we investigated whether miR-155 drove pro-inflammatory cytokine production in our SF system by exploiting the miR-155-LacZ reporter strain of mice, in which replacement of the miR-155 coding sequence by a LacZ cassette generates a non-functional allele [39, 40]: Naïve-SFs from wild type and miR155-deficient (LacZ heterozygotes and homozygous null) mice showed that each of basal and IL-17-, IL-1β- and LPS-stimulated IL-6 and CCL2 release was substantially dependent on miR-155 expression (**Fig 4G and 4H**). Consistent with this, enforced expression of miR-155 via transfection with miR-155-5p mimic, increased basal release of IL-6 and CCL2 by SFs from each of Naïve, CIA and CIA-ES-62 mice and this was associated with profound suppression of SOCS1 (**Fig 4I–4K**), as previously reported for the association between high levels of miR-155 and IL-1β and TNFα in peripheral blood from RA patients [41]. However, this acute treatment with exogenous miR-155 did not impact on the expression of DNMT1 in any of the cohorts of SFs (**Fig 4L**), reflecting the finding that its enforced expression did not convert either Naïve- or ES-62-CIA- SFs to the hyper-responsive CIA phenotype, at least in terms of IL-6 and CCL2 release (**Fig 4I and 4J**), but rather appeared to globally enhance cytokine release, perhaps in part by acutely suppressing SOCS1 that can target TLR signalling [15]. Surprisingly, however, these studies additionally showed that rather than preventing DNMT1 downregulation, ES-62 appeared to further reduce its expression at the mRNA level and again, this was not impacted by enforced expression of miR-155 (**Fig 4L**).

### Hypo-responsive ES-62-CIA-SFs exhibit a distinct global DNA methylation profile to Naïve- and CIA-SFs

**Fig 5:**
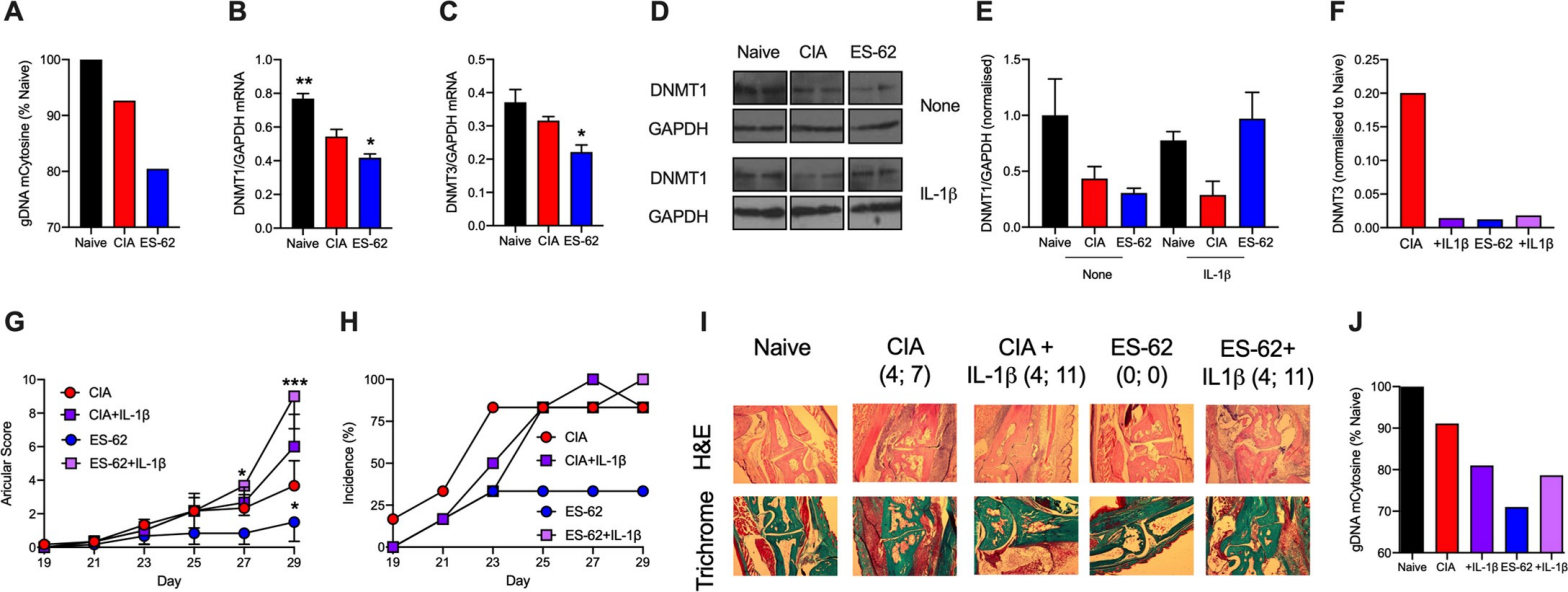
ES-62 induces a CIA-SF phenotype distinct from Naïve-SFs. SFs from Naïve, CIA and ES-62-CIA mice were assessed for their levels of (**A**) global DNA methylation and DNMT1 (**B**) and DNMT3 (**C**) mRNA expression, where data are mean values ± SEM, n = 3 independent cultures and *p<0.05 and **p<0.01 are relative to CIA-SFs. (**D**) Western blot analysis of DNMT1 expression in independent cultures incubated in medium alone or containing IL-1β for 24 h and the data (means ± range) quantitated by ImageJ software presented (**E**) as DNMT1/GAPDH ratios normalized to the “Naïve-None” controls. Pooled data from 6 independent cultures (normalized to Naïve controls) showed relative DNMT1 expression to be: Naïve, 1 ± 0.18; CIA, 0.24 ± 0.10 and ES-62, 0.26 ± 0.06, where ***p<0.001 for Naïve versus CIA or ES-62. (**F**) SFs from CIA- and ES-62-CIA mice were treated with medium or medium containing IL-1β for 24 h and the levels of DNMT3 mRNA determined and normalized to Naïve controls. Throughout, SFs were pooled from individual mice to generate representative cohorts with articular scores: (**A and F**) CIA, 4.75 ± 1.31, n = 8 and ES-62-CIA, 0, n = 6; (**B-E**) CIA, 3.66 ± 1.5, n = 6 and ES-62-CIA, 1.5 ± 1.15 n = 6. (**G-J**) CIA was induced in mice treated with PBS (CIA) or ES-62 on days -2, 0 and 21 and then with PBS or IL-1β (1 μg/dose) twice-weekly from day 21 and articular scores (**G**) and incidence of pathology (**H**) monitored. The experiment was terminated prior to full pathology being established in the CIA control cohort due to the severity of joint disease in the IL-1β treatment groups. (**G**) Articular scores are presented as mean values ± SEM with n = 6 mice/group where *p<0.05 or ***p<0.001 are relative to the CIA group and (**H**) incidence represents the % mice displaying scores >1. (**I)** Representative sections of joint pathology displaying both the score of the joint shown and total articular score of the mouse presented. (**J)** global methylation status of SFs from the indicated cohorts of this CIA ± IL-1β model.

The DNMT1 mRNA data suggested that ES-62 does not simply prevent epigenetic rewiring of Naïve-SFs to the pathogenic, hyper-responsive CIA phenotype. Supporting this, rather than simply maintaining or restoring the levels of DNA methylation observed in Naïve SFs, we found that exposure to ES-62 induces further global DNA hypomethylation relative to CIA-SFs (**Fig 5A**). Perhaps reflecting this, whilst rewiring of SFs from CIA mice was associated with reduced levels of DNMT1, but not DNMT3 (increasingly seen as also modulating the DNA methylome landscape [42]), the more pronounced remodelling of the methylome seen with CIA-ES-62 SFs was accompanied by downregulation of both DNMT1 and DNMT3 mRNA (**Fig 5B and 5C**). Further support that ES-62 does not simply maintain the Naïve-SF phenotype is provided by Western blot analysis confirming that ES-62 did not prevent the downregulation of DNMT1 expression associated with the transformation of Naïve to hyper-responsive SFs in CIA (**Fig 5D and 5E**).

Chronic exposure to pro-inflammatory cytokines seems to be required to achieve epigenetic rewiring of the Naïve- to hyper-responsive SF phenotype (**Fig 2**; [43,44]). However, acute exposure may be sufficient to trigger rewiring, at least in terms of modulation of DNMT1 expression [43,44] and consistent with this, acute treatment with IL-1β appeared to slightly reduce DNMT1 expression in Naïve- and CIA-SFs. Surprisingly, however, such treatment appeared to reverse the profound downregulation of DNMT1 observed in ES-62-CIA-, but not CIA-, SFs (**Fig 5D and 5E**): such differential effects of IL-1β on DNMT1 expression were associated with reduced expression of IL-1R (but not TLR4 or MyD88) mRNA levels in CIA- relative to Naïve- and ES-62-CIA-SFs (**S2J–S2L Fig**). By contrast, IL-1β strongly reduced the level of DNMT3 mRNA pertaining in CIA-SFs to the low level found in ES-62-CIA SFs, which were not further modulated by this acute cytokine challenge (**Fig 5F**).

To investigate the physiological relevance of these findings, we determined the effect of administering (recombinant) IL-1β to mice undergoing CIA (**Fig 5G–5I**): as expected, IL-1β promoted development of severe CIA, with SFs derived from CIA+IL-1β animals exhibiting hyper-MMP responses (**S2M and S2N Fig**) that likely contribute to the enhanced joint damage observed in these mice. Further supporting a role for this cytokine in SF pathogenesis, co-treatment of CIA-mice with IL-1β abolished the ability of ES-62 to protect against development of joint inflammation and destruction, with these animals exhibiting the exacerbated disease seen with CIA-mice treated with IL-1β alone (**Fig 5G–5I**). Of note therefore, SFs from the various treatment groups exhibited stably-modulated global DNA methylation profiles (**Fig 5J**) broadly in keeping with the patterns of DNMT1 expression determined following acute challenge of the various cohorts of SFs, with or without IL-1β (**Fig 5D and 5E**). Thus, exposure to IL-1β *in vivo* induced some additional DNA hypomethylation in CIA-SFs, presumably further promoting de-repression of genes associated with pathogenesis such as MMP-9 and -13. By contrast, *in vivo* treatment with IL-1β resulted in a relative DNA hypermethylation of ES-62-CIA-SFs, potentially reflecting silencing of genes associated with ES-62-protection against CIA.

### ES-62-CIA-SFs constitute a novel CIA-SF phenotype

**Fig 6:**
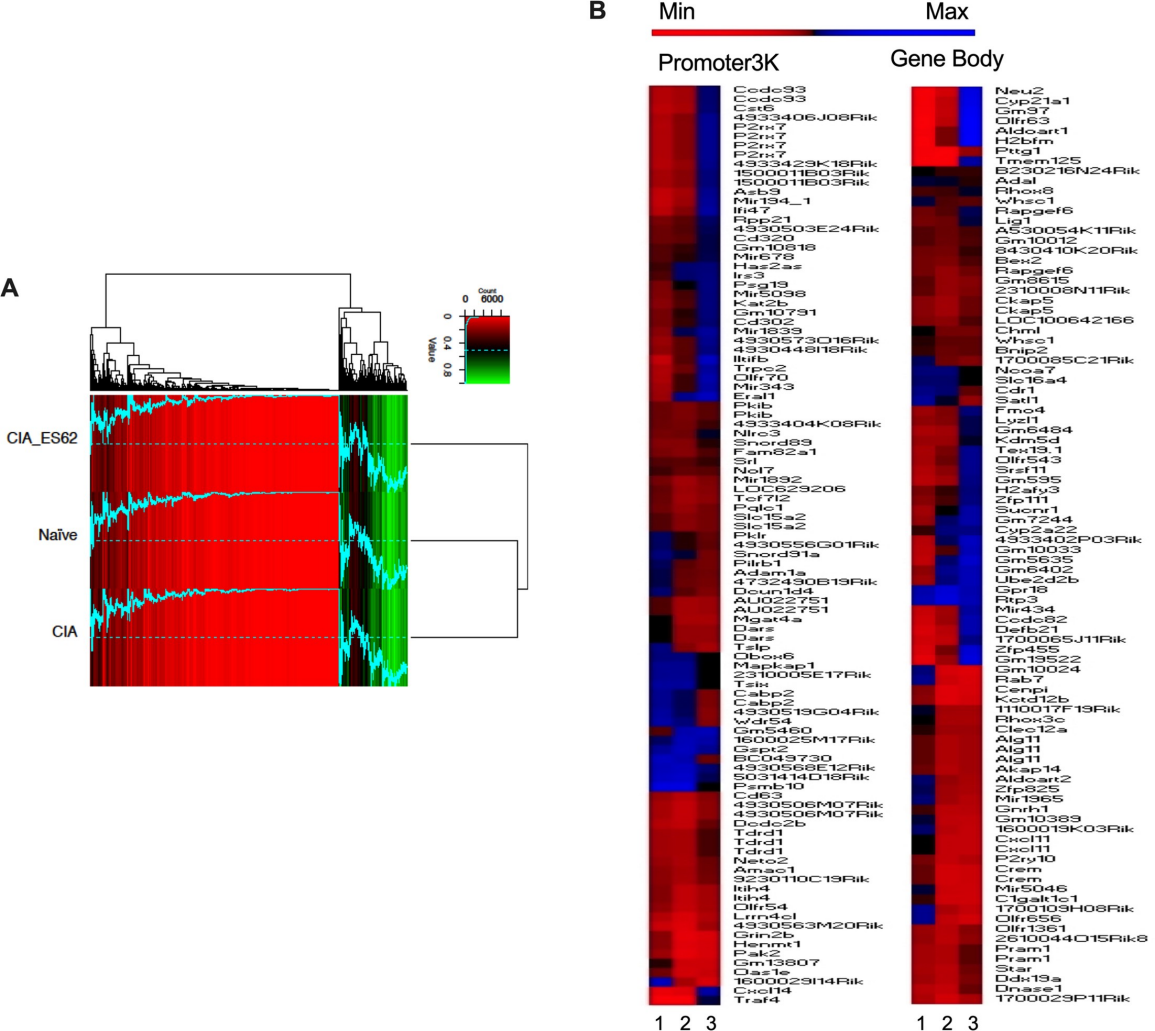
ES-62 induces a DNA methylation phenotype distinct from that of both Naïve- and CIA-SFs. RRBS DNA methylome analysis was performed on SFs from joints representative of the Naïve, CIA (articular score each 3 or 4) and ES-62-CIA (articular score each 0 or 1) cohorts. **(A)** A heatmap of methylation percentages of “Promoter2K” regions (0–2,000bp upstream of TSS) for all cohorts, hierarchically clustered using the Euclidean distance metric. Bright red indicates 0% methylation, black indicates 50% methylation, and bright green indicates 100% methylation. The blue line corresponds to the methylation percentage of the respective regions, thus facilitating visual comparison of methylation percentages across samples (the dotted line represents 50% methylation for reference). (**B**) Methylome analysis of SFs from Naive, CIA- and ES-62-CIA mice showing Heat Map analysis of the top 100 differentially methylated loci in the Promoter3K (left panel) and Gene Body (right panel) regions of total genomic DNA. In each case, hypo (red) or hyper (blue) methylation in SFs from PBS- or ES-62-treated CIA-mice relative to SFs from healthy, naïve mice is shown in lanes 1 and 2 respectively, whilst that of SFs from ES-62- relative to PBS-treated CIA-mice is shown in lane 3.

**Fig 7:**
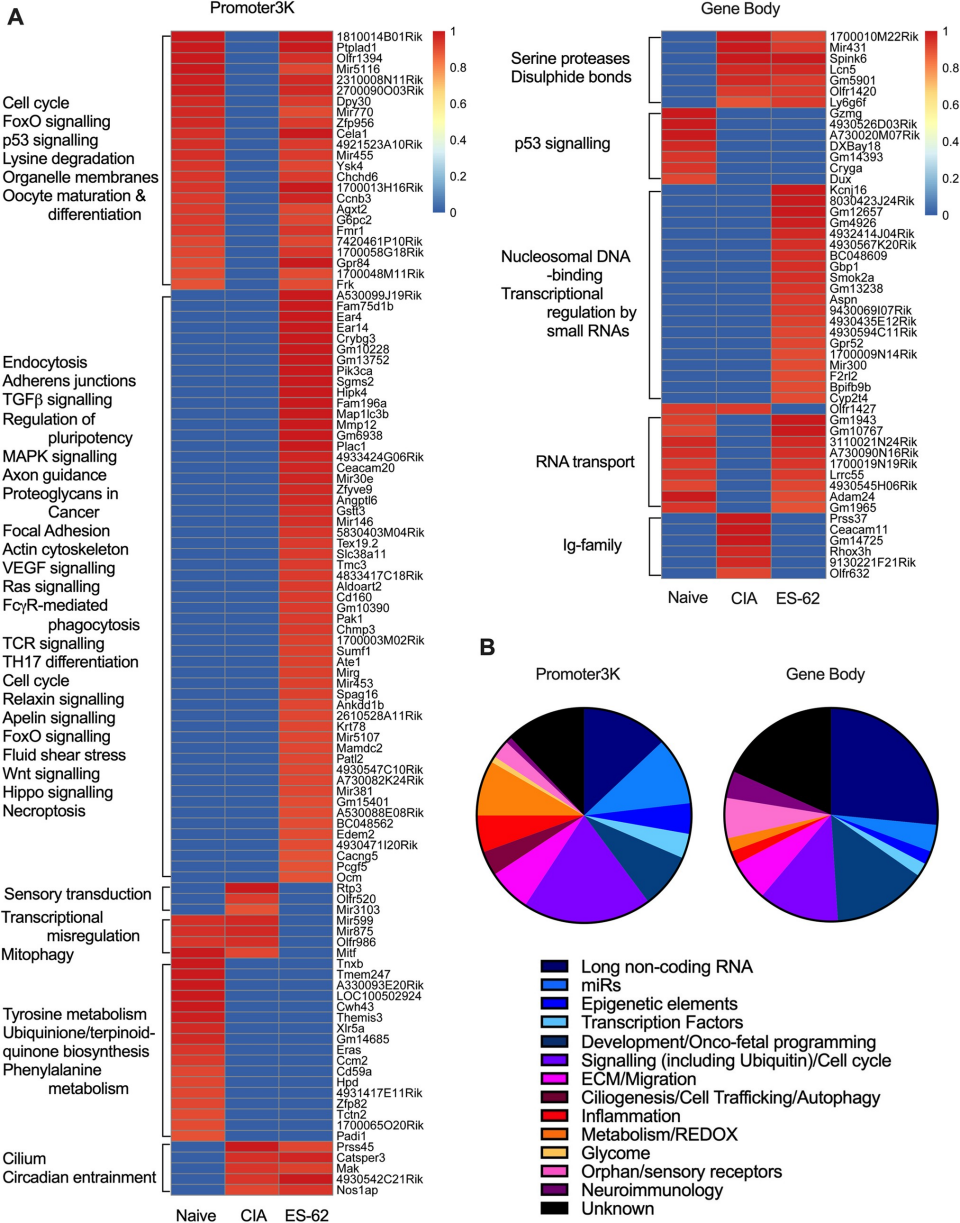
Binary analysis of the DNA methylome indicates differential silencing and activation of genes in SFs from Naïve, CIA and ES-62-CIA mice. Hierarchical clustering heatmaps (**A**) were constructed for the Promoter3K and Gene Body regions analysing genes which were either essentially fully methylated (>0.9) or demethylated (0) at these sites in one or more of the treatment groups. The heatmap clusters are annotated with predicted KEGG pathway interactions and the functional classification of genes constituting these binary DNA methylation signatures differentially targeted in pathogenic (CIA) and protective (ES-62) rewiring of SFs are summarized in the pie-charts according to the accompanying colour code **(B)**.

Collectively our data suggest that rather than simply blocking CIA-associated transformation of Naïve-SFs, ES-62 further epigenetically modifies these to a novel “resolving” CIA-SF phenotype. To provide proof of principle of this, we subjected SFs representative of the Naïve-, CIA- and ES-62-CIA-SF cohorts to Reduced Representation Bisulphite Sequencing (RRBS) and bioinformatic analysis (**Figs 6, 7, S3 and S4**) and explored whether they exhibited distinct DNA methylome profiles. Certainly, hierarchical clustering analysis of the methylation percentage of Promoter regions revealed the profile of ES-62-CIA-SFs to be distinct to those of both the (more closely related) Naïve and CIA groups (**Fig 6A**). Moreover, differential DNA methylation analysis (top 100 sites) of the Promoter3K (3000 bp upstream of transcription start site [TSS]) and Gene Body (start of 5’UTR to end of 3’UTR) regions indicated that ES-62 only acted to maintain the “Naïve” methylation status in a limited number of genes and instead, differentially induced both hypo- and hyper-DNA methylation of various genes relative to their Naïve- and CIA-SF counterparts (**Fig 6B**). The functional outcome of such changes in methylation status is not straightforward to understand however, as whilst hypermethylation at the promoter is associated with gene silencing, within the gene body it appears to correlate with gene expression and regulation of splice variants [45–48]. Thus, for example, visualization of the differential profiles of CpG methylation at exon 3 of the ES-62 target, MyD88 (**S3A Fig**) shows hypermethylation, and hence potential upregulation, in CIA-SFs relative to that in naïve and ES-62-CIA-SFs. By contrast, and consistent with our functional data, CIA-SFs display hypermethylation (gene silencing) within the Promoter and TSS regions of the SOCS1 gene relative to SFs from both Naïve and ES-62-CIA mice (**S3B Fig**).

To investigate whether the differential DNA methylation exhibited by the three cohorts could provide insight into the mechanisms underpinning the generation of their functionally distinct SF phenotypes, we performed pathway enrichment analysis using String and KEGG databases. As a first step to identifying cohort-specific signatures, we adopted an “all or nothing” binary approach, examining sites that were either essentially fully methylated (>90%) or demethylated (0%) at the Promoter3K or Gene Body regions in one or more of the treatment groups (**Figs 7 and S4**). Heatmap analysis (**Fig 7**) revealed the distinct nature of the Naïve-, CIA- and ES-62-CIA-SF phenotypes whilst KEGG analysis of these binary signatures (**Figs 7 and S4**) supported the functional relevance of their differential epigenetic profiles: for example, it highlighted differential promoter silencing of the Ras/MAPkinase, FoxO, Hippo and Wnt signalling pathways that are key to the regulation of cellular processes (stem cell biology/pluripotency, ciliogenesis and intercellular communication, cell proliferation and migration, organ development and size, immunomodulation, tissue repair and regeneration and ageing [49–51]) dysregulated in SF pathogenesis. Cross-mining of these data with genes already implicated in the (SF) pathogenesis of RA (**S1 Table**) further supports the hypothesis that ES-62 does not simply prevent remodelling of SFs during CIA: rather, this analysis suggests that by (additionally) modulating expression of genes (e.g. *miR146*, *Lair1*, *Pak1*, *Pik3ca*, *Map1lc3b*, *Aspn*, *Ccl25*, *Mmp12*, *Spag16 and Adipoq)* implicated in the dysregulation of SF responses, ES-62 may stably rewire CIA-SFs to a distinct and protective “resolving” phenotype that may counter developing and established inflammation and bone destruction in the joint.

### PC-based SMAs mimic the ability of ES-62 to stably rewire SF responses

**Fig 8:**
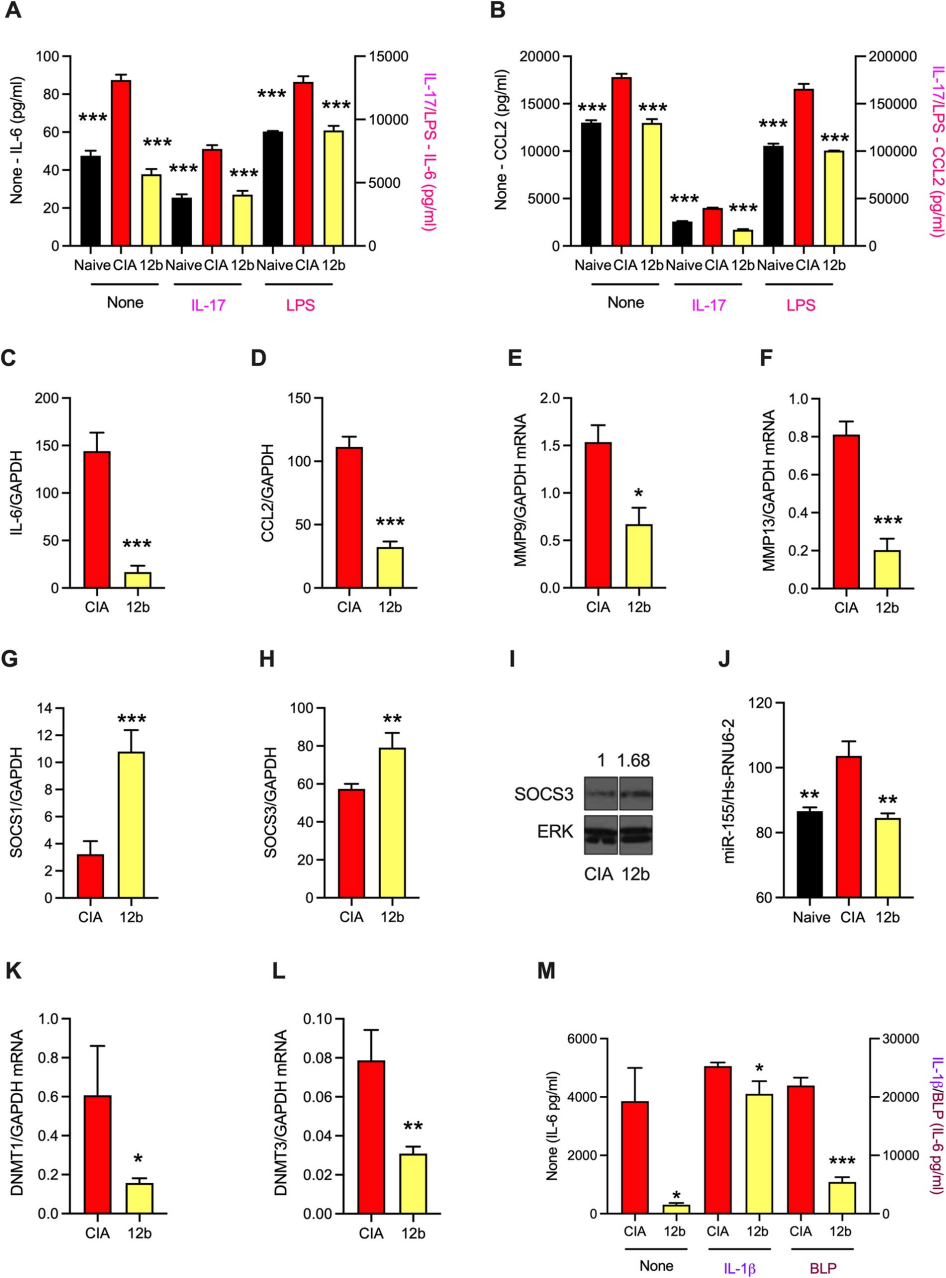
Exposure of CIA-mice to SMA 12b *in vivo* results in SF hypo-responsiveness *ex vivo*. Mice were treated with PBS or 12b (1 μg/dose s/c) at days -2, 0 and 21 of the CIA protocol. Naïve-, CIA- and 12b-CIA SFs (12b) were incubated in medium (None), or medium containing either IL-17 (25 ng/ml) or LPS (1 μg/ml) as indicated for 24 h and IL-6 (**A**) or CCL2 (**B**) release measured. Levels of mRNA (normalised to GAPDH) in the indicated groups of SFs were determined for IL-6 (**C**), CCL2 (**D**), MMP9 (**E**), MMP13 (**F**), SOCS1 (**G**) and SOCS3 (**H**). For SOCS1 (**G**), the CIA control data are the same as those presented in **Fig 1H**. Data are presented as means ± SEM values of 3 independent cultures except for MMP9 and MMP13, where data are means ± SD, n = 3 from a single culture. (**I**) Western blot determination of SOCS3 expression in CIA and 12b SFs is normalised to that of ERK by Image J analysis. (**J**) Naïve-, CIA- and 12b-CIA SFs (12b) were treated with LPS for 6h and levels of miR-155 determined and the data presented as mean ± SEM values for three independent cultures. Levels of DNMT1 (**K**) and DNMT3 (**L**) mRNA (normalised to GAPDH) in CIA and 12b SFs are presented as means ± SD, n = 3 from a single culture. (**M**) From the first appearance of clinical articular score following challenge with collagen II (CII) at d21, mice were randomly allocated to blinded treatments (PBS or 12b, 1 μg/dose s/c) every 3 days until cull. CIA- and 12b-SFs were incubated in medium (None), or medium + either IL-1β (10 ng/ml) or BLP (0.5 μg/ml) for 24 h and IL-6 release measured. Data are mean ± SEM values of 3 independent cultures (each assayed in triplicate). Throughout, SFs were pooled from individual mice to generate representative explant cohorts with articular scores: (**A-L**) CIA, 3.66 ± 1.50, n = 6; 12b, 0.71 ± 0.36, n = 6 and (**M**) CIA, 5.50 ± 0.96, n = 4; 12b, 2.33 ± 0.33, n = 3. For statistical analysis, *p<0.05, **p<0.01 and ***p<0.001 are for the indicated responses relative to the CIA group.

To further investigate the mechanisms underpinning ES-62 rewiring of SFs *and* explore their translational potential, we investigated the role of the PC-moiety of ES-62 in rewiring SF responses. Certainly, PC chemically conjugated to ovalbumin (PC-OVA) or BSA (PC-BSA), but not recombinant ES-62 (which lacks PC), mimics ES-62’s ability to protect against TH1/TH17 autoimmune responses and development of CIA [52, 53], as well as its *in vitro* inhibition of IFNγ production from cytokine (IL-12 and IL-15/IL-18)-stimulated synovial membrane cultures derived from RA patients [52]. Extending the translational potential of ES-62, we have shown that two SMAs (11a and 12b), designed to mimic the ES-62 PC-moiety, mirror its ability to downregulate MyD88 activity and suppress CIA [53–55]. We now show that these SMAs can act directly to modulate IL-6, CCL2 and miR-155 responses of CIA-SFs *in vitro* (**S5A–S5C Fig**). Furthermore, focusing on 12b, we find that *in vivo* exposure of CIA-mice to the SMA (12b-SFs) results in the reduced IL-6, CCL2, MMP9, MMP13 and increased SOCS1 and 3 responses exhibited by such CIA-SFs *ex vivo* (**Fig 8A–8I**). Importantly, in terms of the epigenetic remodelling of SF responses, whilst exposure to SMA 12b *in vivo* is also able to mimic the ability of ES-62 to modulate miR-155 and DNMT1/3 expression in CIA SFs (**Fig 8J–8L**), *in vitro* co-culture with 12b promotes global DNA hypomethylation yet suppresses the IL-6 hyper-responsiveness that would otherwise result from chronic exposure of Naïve SFs to IL-1β (**S5D and S5E Fig**). These actions of 12b are not restricted to prevention of SF transformation as, in in a therapeutic model of CIA [54], the protection resulting from treatment with the SMA was associated with SF hypo-responsiveness, in terms of spontaneous, IL-1β and BLP-stimulated IL-6 release, *ex vivo* **(Fig 8M**).

Collectively, these data indicate that (i) the PC-moiety can mimic a range of ES-62’s actions on SFs; (ii) PC-ES-62/SMAs do not simply block rewiring of Naive SFs to a “pathogenic” phenotype; (iii) PC-ES-62/SMAs can modulate SF responses even when the levels of “pathogenic” cytokines are experimentally maintained in a chronically elevated state (by daily refreshing the levels of IL-1β [and SMA12b] in the *in vitro* chronic inflammation co-cultures) (iv) PC-ES-62/SMAs can exert direct and indirect (via suppression of cytokines like IL-1β and IL-17) modulatory actions on SFs and (v) cross-talk between IL-1β- and PC-ES-62/SMA-driven signals drives further SF epigenetic remodelling to a distinct “protective” phenotype.

## Discussion

Increasing evidence suggests that exposure to pathogens, including parasitic worms, shapes our immune responses and indeed, such “training” has been associated with the epigenetic rewiring of immune system effectors, as well as their progenitors and haemopoietic stem cells. Indeed, although not examined at the level of the methylome, we have previously shown ES-62 (which, based on a protein BLAST search, has homologs in other filarial nematodes including species that parasitise humans, with ~70% identity*)* to induce stable anti-inflammatory phenotypes of dendritic cells (DCs) and macrophages (from bone marrow-derived progenitors) and immunoregulatory B cells in the MRL/Lpr mouse model of SLE [56–58]. Thus, in this context, there are key novel features arising from the current study. Firstly, that a single, defined parasite-derived molecule possesses the capacity to induce stable epigenetic remodelling of target cells to suppress the pathogenic outcomes of chronic inflammation. Secondly, that ES-62 does this, not by simply suppressing the chronic inflammation and subsequent reprogramming of aggressive inflammatory cells, but rather, by additionally directing further cell differentiation to a novel “protective/repair” phenotype.

Interestingly therefore, the suppression of DC maturation, particularly in terms of TLR signalling, by total excretory-secretory products released by the parasitic trematode worm *Fasciola gigantica* (FgESPs) has recently been shown to be associated with changes in the methylome and transcriptome reprogramming [59]. Moreover, the methyl-CpG-binding protein, Mbd2, that acts to coordinate chromatin accessibility and hence, reprogramming of gene expression in response to DNA methylation, is key to the ability of DCs to drive Th2 responses to helminths and allergy [60] and also plays a role in CD11c^+^ DC/monocyte-epithelial cell crosstalk in gut inflammation [61].

Excitingly in terms of the wider context, given that ES-62 also stably rewires innate and adaptive immune responses, such reprogramming of haemopoietic and stromal cells suggests that helminths have evolved integrated inflammation-resolving and repair/regeneration strategies to counter the chronic inflammation and tissue pathology they would otherwise elicit. Consistent with this, it has recently been reported that another parasitic trematode, *Schistosoma mansoni*, induces hepatocyte DNA hypomethylation that is associated with reduced tissue pathology and granuloma formation in the acute phase of infection in mice [62], supporting the idea that epigenetic changes induced by helminth infection appear to be indirectly important in promoting long-term helminth survival. Interestingly, such epigenetic remodelling was impacted by the inflammatory context, which modulated the (reciprocal) epigenetic changes occurring in both the host hepatocytes and the worms to determine the levels of liver pathology [62]. The potential importance of the evolution of such complex host-worm epigenetic regulatory networks is highlighted by evidence that CD4 T cells, from children recently exposed to tuberculosis and infected with the related *Schistosoma haematobium* showed profound differential DNA methylation, relative to those from uninfected children, that was associated with TH2-skewing of tuberculosis (TB)-specific responses away from the protective TH1/IFNγ phenotype [63]. Moreover, the remodelled DNA-methylation signatures and defective TB-specific TH1 responses were maintained for up to at least 6 months following successful deworming of *S*. *haematobium* [63]. Furthermore, intriguingly, emerging evidence suggests that the offspring of chronically-infected mothers display epigenetically rewired immune responses that potentially shape their reactions to other pathogens (bacterial and viral infections) and vaccines [64, 65]. Thus, understanding these interactive epigenetic networks has important implications not only for how to develop strategies to counter the rise in prevalence of chronic inflammatory (allergic and autoimmune) conditions, increasingly associated with eradication of helminths, but also to combat pathogens in a co-infection setting.

**Fig 9:**
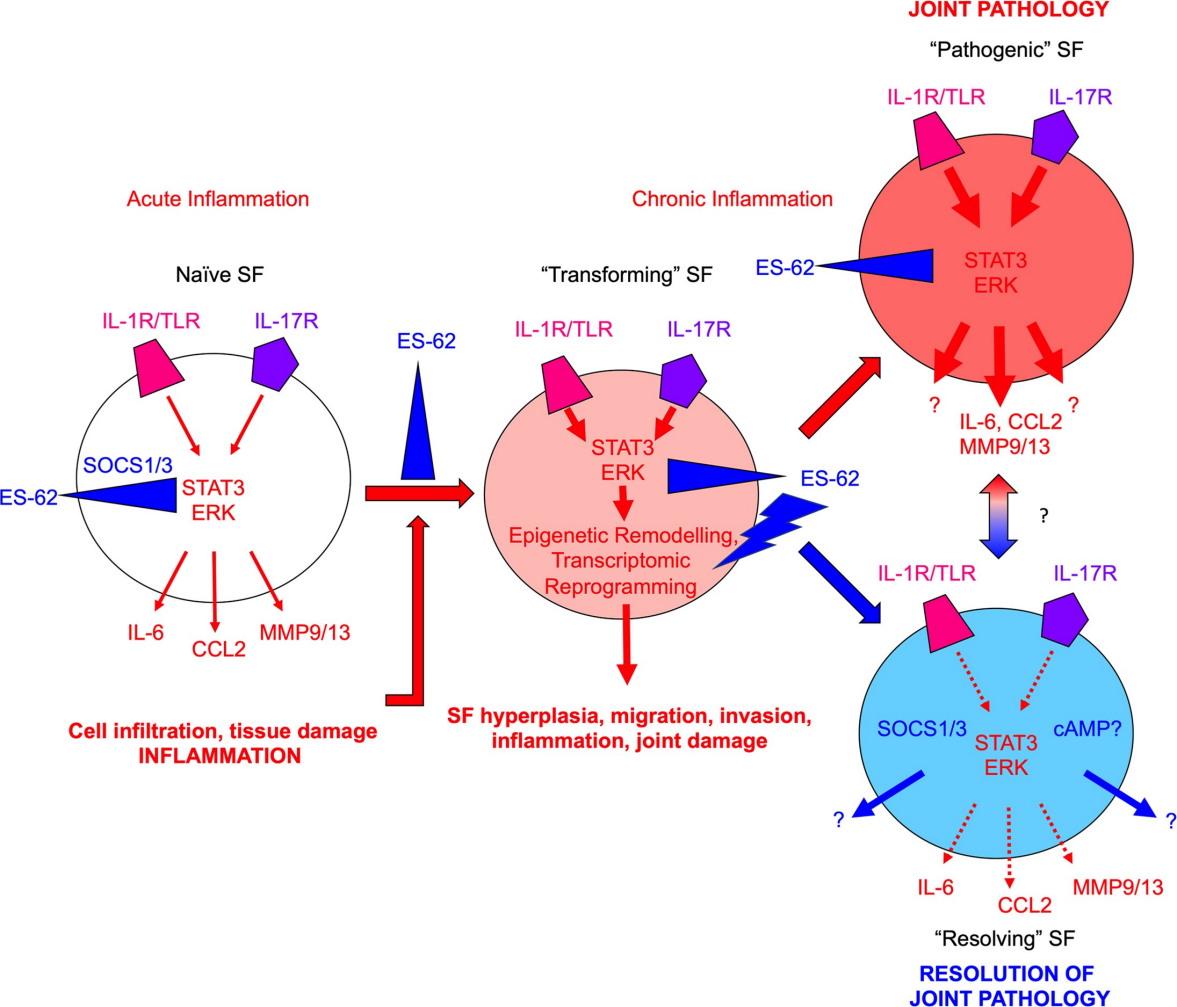
Model of ES-62 action in SFs during CIA. Healthy SFs (Naïve) respond to acute pro-inflammatory signals (e.g., IL-1β/IL-17) in the joint microenvironment resulting in release of pathological mediators such as IL-6, CCL2 and MMP9/13, which cause cell (neutrophils, macrophages, lymphocytes) infiltration and tissue damage, resulting in chronic inflammation. In addition to perpetuating joint inflammation and damage, chronic inflammation drives rewiring of the epigenetic landscape and consequently, reprogramming of the SFs to a “pathogenic” aggressive hyperplastic, migrating and invasive phenotype that, via its hyper-responsiveness to environmental cues, plays a key role in joint pathology and destruction (all pathogenic signals denoted by red arrows with imprinted hyper-responsiveness represented by increased arrow size and redness of SF). ES-62 can disrupt this process at multiple points: thus, it can inhibit (blue blocking symbol) the (i) initial pro-inflammatory signalling to suppress acute inflammation; (ii) inflammatory signals generated by infiltrating cells and tissue damage to suppress induction of chronic inflammation; (iii) signalling associated with chronic inflammation to suppress transformation of SFs to an aggressive hyper-responsive phenotype and (iv) pathogenic signals in aggressive SFs during established disease. Consistent with our findings that ES-62 does not simply maintain/restore the Naïve-SF phenotype, it also exhibits positive actions (blue lightning bolt symbol), specifically inducing epigenetic remodelling (perhaps of an intermediate anti-inflammatory SF phenotype, generated by inhibition of pathogenic ERK and STAT3 signalling) that reprograms the cell to an inflammation-resolving and tissue repair phenotype. The potential ability of the “resolving” ES-62-CIA-SFs to also directly influence the behavior of “pathogenic” SFs in the joint during established disease is represented by the red-blue gradient arrow.

In terms of autoimmune arthritis, our studies also shed mechanistic light on how both acute and chronic interactions within the inflammatory microenvironment of the joint during induction and progression of CIA shape SF responses, resulting in remodelling of their epigenetic landscape in terms of DNA methylation and reprogramming to a hyperinflammatory, joint destructive cell phenotype (**Fig 9**). This stepwise process is characterised by increased expression of cytokines and growth factors (and their receptors), signalling molecules (e.g., Wnt, RasMAPkinase, FoxO pathways), adhesion molecules and extracellular matrix components and proteases [4,6–8]. Reflecting their high levels of expression in the CIA joint [11,12,54], IL-1β and IL-17 are implicated in driving such reprogramming via DNA hypomethylation induced by downregulation of DNMT1 [43] and evidenced by its recapitulation *in vitro*, following chronic exposure of Naïve-SFs to these pro-inflammatory factors and the DNMT1 inhibitor 5-azacytidine (5-aza). Moreover, reflecting a convergent upstream signalling pathway, both the acute (cytokine and MMP production) and chronic (remodelling to a stable aggressive phenotype) responses to these pathogenic cytokines are dependent on ERK and STAT3 signalling. Consistent with our previous studies demonstrating that the protection afforded by ES-62 against CIA was associated with the suppression of the hyper-inflammatory phenotype of CIA-SFs [12], we found the acute production of cytokines and MMPs by this stably imprinted “resolving” phenotype to be reduced to levels more typical of those of Naïve-SFs and this hypo-sensitivity was associated with a corresponding suppression of ERK and STAT3 signalling. Surprisingly therefore, these stably protective actions were not due to ES-62 simply preventing the transformation of SFs to the “pathogenic” hyper-responsive phenotype but rather, reflected further epigenetic remodelling associated with a distinct hypo-responsive CIA-SF phenotype (**Fig 9**). Thus, given the functional differential outcomes of DNA methylation of Promoter versus Gene Body regions [45–48] and our finding that ES-62’s effects reflect even more profound global DNA hypomethylation than that observed in CIA-SFs, these studies suggest that utilizing interpretation of the global DNA methylation status of RA patients as a biomarker of disease is a strategy that should be treated with caution.

The ability of IL-1β and IL-17 to drive key features of SF pathogenicity [25,66,67], and their suppression, both systemically and in the joint, by ES-62 and its SMAs [11,12,53,54], underscores the importance of fully understanding the mechanisms underpinning these interactions to develop strategies for therapeutically promoting re-establishment of joint homeostasis. Certainly, ES-62 (and SMAs based on its active PC-moiety) can act directly to suppress “inflammatory” SF responses, presumably (as with their effects on a wide range of cell types) via TLR4 to suppress MyD88 signalling [11,53–55,57,68–70]: indeed, our preliminary data with gene-deficient SFs suggest that the direct inhibition of LPS-stimulated CCL2 production by SFs reflects targeting of MyD88 by the SMAs. These direct actions corroborate our previous reports that, whilst acute exposure to ES-62 only marginally impacts on the steady-state gene expression profile of naïve macrophages *in vitro*, it inhibits LPS-stimulated IL-6 and TNFα production and modulates acute LPS-induced changes in gene expression in synovial membrane cell cultures from autoimmune RA patients, transcriptional reprogramming of “inflamed” cells by ES-62 that again generates a profile distinct from that of either the untreated or LPS-stimulated synovial membrane cells [71,72]. Moreover, its lack of modulation of basal responses of Naïve-SFs is consistent with our observation that ES-62 does not appear to impact in any way on the joints of naive CIA-prone DBA/1 mice [10] and further supports our hypothesis that ES-62 acts to homeostatically reset the aberrant, high levels of inflammation pertaining in chronic inflammatory disorders to "normal" healthy levels, rather than inducing a generalised suppressed phenotype [56]. However, as ES-62 (and its SMAs) also acts to reset immunoregulation and reduce and/or resolve elevated levels of pathogenic cytokines in CIA [10–12,53,54], collectively these data may suggest that it counteracts the rewiring of SFs in CIA by direct and indirect mechanisms, involving both the suppression of inflammatory mediators and inhibition of their downstream signalling. Such a dual-pronged approach would result in ES-62 maintaining certain Naïve-SF methylation signatures that are anti-inflammatory and/or promote inflammation resolution. However, ES-62 does not simply prevent induction of the hyper-responsive SF phenotype, as a result of its suppression of pathogenic IL-17-mediated ERK and STAT3 signalling during CIA (**Fig 9**). Rather, it appears to drive further (arthritis-associated) epigenetic changes, such as the Promoter3K hypermethylation (silencing) of PAK1, a kinase identified as a key driver of SF migration and invasion in RA [73] and one of our Binary Signature genes. Thus, the ultimate generation of “resolving” ES-62-CIA-SFs could proceed in a stepwise manner, with novel protective “arthritis-specific” epigenetic changes resulting from the signalling crosstalk amongst cues from ES-62 and pathogenic joint microenvironmental factors, in a “primed” intermediate SF phenotype (**Fig 9**).

Although the processes by which ES-62 directly suppresses the ERK and STAT3 signalling underpinning SF transformation have not been fully determined, we have previously shown it to suppress ERK by both uncoupling upstream regulators and promoting MAPK phosphatase activity in innate and adaptive immune system cells [74]. We now also show that ES-62 upregulates the important negative feedback regulators of inflammatory signalling, SOCS1 and SOCS3, that are also likely to impact on IL-1β/IL-17 pathogenic pathways in SFs. Thus, for example, SOCS1 can suppress IL-6 (indirect effector of IL-1β) and IL-1R/TLR (via targeting of Mal, IRAK1, Traf6 and NF-κBp65) signalling [15] and both SOCS elements can directly negatively regulate JAK-STAT signalling, with typically (IFN-driven) STAT1 signalling being inhibited by SOCS1 [75] and (IL-1β-, IL-6- and IL-17-driven) STAT3 signalling suppressed by SOCS3 [76]. Of relevance, miR-155 has been shown to negatively target SOCS1 [15], promoting the pathogenic IFNγ signalling [75,77,78] in CIA that is targeted by ES-62 [71]. Similarly, miR-146, which is found at elevated levels in RA-SFs and in response to key pathogenic mediators (e.g., IL-1β, IL-6, IL-17 and TNF-α), upregulates IRAK1 and Traf6 to enhance inflammatory signalling [25,26]. Thus, the finding that ES-62 reduces miR-146 and -155 levels suggests that this might provide an acute and dynamic mechanism by which it maintains levels of SOCS1 and SOCS3 during CIA to regulate and resolve pathogenic SF responses elicited by IL-1R/TLR and cytokine signals chronically generated in the inflamed joints. Perhaps surprisingly, given the pathogenic role of the STAT3 pathway in IL-1β/IL-17 signalling and development of CIA, as well as its targeting by ES-62, enrichment of JAK-STAT pathways was rather weak in the “all or nothing” Binary Signatures. However, closer analysis of the data reveals more subtle methylation changes in these pathways (potential silencing of arthritis-promoting JAK1, STAT3, STAT5b and PIAS3 elements and activation of -antagonistic STAT6 and PIAS1) that are consistent with the observed functional SF phenotypes [79,80], although the significance and robustness of these data await confirmation by higher power analysis of the treatment groups in terms of individual mice.

Support for the future translational development of the differential DNA methylation SF signatures associated with naïve, CIA and ES-62-CIA mice is provided by recent mathematical modelling of differential gene expression metadata from a number of studies in healthy and RA synovial tissue [81]. This identified a gene signature panel representing an interactive biological network of hub (STAT1, RAC2 and KYNU) proteins and effector molecules (PEPD receptor and NR4A1, MEOX2, KLF4, IRF1 and MYB transcription factors and miRs-146a, -299, -3659, -6882 and -8078) that provides a platform for validation of pathogenic mechanisms and potentially, a predictive tool for diagnosis and development of effective treatment strategies for RA: apart from KYNU and miR-299 (miRs-3659, -6882 and -8078 were not identified in our methylome study), our proof-of-concept methylome analysis suggests that these “signature” genes may be differentially methylated in naïve-, CIA- and ES-62-CIA-SFs. Likewise, very recently [82], an integrated epigenetic study (histone marks, open chromatin, transcriptomics and DNA methylation) identified 8 epigenetic clusters that differentially modified in healthy and hyper-responsive SFs (from both humans and mouse models). Although different mouse models to CIA (hTNFtg and G6PI-induced arthritis models) were analysed, essentially all of the 52 genes exhibiting differential DNA methylation, and clustered on the basis of their involvement in the proliferation, migration and cell-cell interaction processes involved in the pathogenic transformation of RA SFs, appeared to be differentially methylated amongst our Naïve, CIA and CIA-ES-62 groups, albeit in some cases these changes were subtle and all require statistical validation. Of note, given the key role proposed for upregulation of Lasp1 and its regulation of adherens junction formation in SF transformation and joint damage, our methylome data were consistent with increased expression of Lasp1 in CIA-SFs being countered by exposure to ES-62 *in vivo*.

Thus, understanding how the complex epigenetic networks underpinning SF transformation are subverted by ES-62 action, may allow for development of novel and safe joint-targeted therapeutic interventions. For example, ciliogenic pathways appear to be promising targets of ES-62 as cilia coordinate crosstalk amongst the JAK-STAT, MAPkinase, Akt, FoxO, Hippo and Wnt pathways [83–85] to generate the complex signalling networks required to integrate the critical homeostatic cellular processes (pluripotency and differentiation; organ development and size; cell survival and proliferation, migration and communication; inflammation; mechano-sensing, tissue repair and regeneration [49,50,86]) that become disrupted during pathogenic SF transformation. Certainly, cilia-associated functions predominated in the genes exhibiting fully methylated Promoter3K regions in the ES-62-CIA-SF cohort, with ES-62 uniquely hypermethylating the promoters of genes implicated in ciliogenesis (MapKap1 [Sin1 subunit of mTORC2], Inpp5b, DynII2, Chmp3, Pik3ca) and migration and interaction with the ECM (Cela1 [chymotrypsin-like elastase family member 1], Ate-1, Spag16). Moreover, we have previously shown ES-62 to strongly induce negative regulators (WIF1, AMOTL2 and CRYBB2) of Wnt signalling in RA synovial membranes, *in vitro* [72]. Critically, ES-62 silencing (promoter hypermethylation) of these genes associated with ciliogenesis and its regulation of signalling and differentiation is not a reversion to the Naïve-SF phenotype. Interestingly, however, many of the genes displaying fully methylated Promoter3K regions in the Naïve-SF cohort are components of Ubiquitin-proteosome pathways, some of which have also been implicated in the regulation of ciliogenesis [83]. This could suggest that ES-62 exploits a “variation on a theme” to correct dysregulation of ciliogenesis and underscores the potential of ES-62, driving rewiring of a unique and “resolving” phenotype of SF in mice undergoing CIA.

In conclusion, during patency, the ability of ES products to reprogramme haemopoietic and stromal cell responses likely contributes substantially to parasite survival and thus, further understanding of this underexplored feature of the co-evolution of the host-parasitic worm relationship opens up potential new targets to combat these pathogens safely, particularly in a co-infection setting. Crucially, given our recent findings that ES-62 can promote both health- and life-span in male mice undergoing obesity-accelerated ageing [87], identification of the key (tissue-specific) molecular signatures involved could also ultimately be exploited therapeutically, not only to counter the rise in prevalence of chronic inflammatory (allergic and autoimmune) conditions, increasingly associated with eradication of helminths, but also to develop regenerative strategies to promote healthspan and reduce the socio-economic impact of our ever-increasing ageing populations.

## Materials and methods

### Ethics statement

Mouse maintenance and experimental procedures and protocols were approved by the University of Glasgow Animal Welfare and Ethical Review Board and all studies were performed in accordance with UK Home Office Project and Personal Licences (PPL60/26532, I675F0C46, PIL70/26532), following the “principles of laboratory animal care” (NIH Publication No. 86–23, revised 1985).

### Collagen-induced arthritis (CIA) model

Male DBA/1 (Envigo, UK) mice were housed (2-4/cage) in the Central Research Facility of the University of Glasgow at 22–24°C and 40–50% humidity under specific pathogen-free conditions according to treatment group, as previous studies identified a role for the gut microbiome in ES-62-protection against CIA [10]. Mice were maintained under a 12 h light/dark cycle and *ad libitum* access to water and chow (CRM-P; SDS, UK; Oil, 3.36%; Protein 18.35%; Fibre, 4.23%: Sugar 3.9%; Atwater fuel energy from Oil, 9.08%; Protein, 22.03%: Carbohydrate, 68.9%) plus 150 ppm Fenbendazole. For the CIA model, male DBA/1 mice (8-weeks old) were injected intradermally on day 0 with 100 μg Bovine Type II collagen (CII; MD biosciences) in complete Freunds adjuvant (CFA; MD biosciences) just above the tail base. On Day 21, mice were administered 200 μg of type II collagen in PBS via intra-peritoneal injection [71]. Mice were monitored every two days for signs of arthritis as described previously [71], being determined according to the following articular scores: 0 = normal; 1 = digit(s) involvement; 2 = erythema; 3 = erythema and swelling; 4 = extension/loss of function and in addition, by calliper measurement of hind paw swelling (POCO 2T, Kroeplin Längenmesstechnik). Where indicated, ES-62 (2 μg in PBS, prepared endotoxin-free and purified as previously described [71]) or 12b (1 μg in PBS, prepared as described previously [54]) was administered subcutaneously in the scruff on days -2, 0 and 21. Analysis of all the mice (including CIA-mice with no pathology) from the models contributing to these studies (see Figure legends for details of individual experiments) shows that SFs were obtained from CIA or ES-62-CIA mice with overall mean articular scores of 3.91 (median of 3) ± 0.32, n = 103 and 2.15 (median of 1) ± 0.48, n = 39, respectively. Thus, exposure to ES-62 significantly (p = 0.0004) suppresses pathology by some 45% overall, as indicated by 2-tailed Mann-Whitney analysis. Similarly, SFs were obtained from CIA or 12b-CIA mice with overall mean articular scores of 2.89 (median of 2) ± 0.72, n = 18 and 1.23 (median of 0) ± 0.49, n = 18, respectively. Thus, exposure to 12b significantly (p = 0.0483) suppresses pathology by some 57% overall, as indicated by 2-tailed Mann-Whitney analysis.

SFs were isolated and cultured according to the protocol of Armaka *et al* as described previously [88]. Briefly, crushed joints were treated with 1 mg/ml collagenase IV (*Clostridium histolyticum*; Sigma) at 37°C for 1 hour and the released cells resuspended in DMEM (Lonza), supplemented with 10% FBS, 2mM L-glutamine, and 50 units/ml penicillin and 50 μg/ml streptomycin, and plated in 100 mm dishes. After 24 hours, non-adherent cells were removed and the cells passaged (medium replaced twice weekly) and expanded for 3 to 4 weeks before analysis. The SF phenotype of the explant cultures (CD54^+^CD106^+^CD90.2^+^) was confirmed by flow cytometry [12] (**S6 Fig**).

Analysis of *in vivo* hypoxia status of SFs was determined using Pimonidazole HCl (dose of 60 mg/kg; Hypoxyprobe^_^1 plus kit protocols) injected intra-peritoneally 24h prior to cull [89]. Joint cells were extracted and then fixed in chilled 70% ethanol and stored at -20°C for 24 hours. Cells were next resuspended in PST (PBS with 4% serum and 0.1% [v/v] Triton X-100) and stained using the kit FITC-conjugated Mab1 (diluted 1:1000) for 2 hours at 37°C prior to analysis by flow cytometry. Likewise, the vasculature permeability occurring *in vivo* was assessed following intravenous injection (200 μl) of Evans blue dye (Sigma; 0.5% in PBS) in the mouse lateral tail vein 30 min prior to cull [90]. For histochemistry, following fixation and decalcification of bones, joints were paraffin-sectioned and stained with H&E or Trichrome solutions as described previously [12].

### Flow cytometry

SFs (0.5 x 10^6^) were stained with the fixable viability dye eFluorⓇ 780 (1 μg/ml; eBioscience) prior to blocking Fcɣ receptors and incubation with the indicated primary (2 μg/ml each of anti-CD54-PE [YN1/1.7.4; Biolegend]; anti-CD90.2-PerCP [30H12; Biolegend]; anti-CD106-PE-Cy7 [429; eBioscience] or appropriate isotype control) antibodies. For intracellular staining of signalling molecules, cells were first stimulated as indicated prior to permeabilization with methanol and then ERK protein (ERK), dually phosphorylated ERK (pERK), STAT3 protein (STAT3) or phosphorylated (pSTAT3) expression detected by the relevant rabbit antibodies (all Cell Signalling Technology) and secondary FITC-conjugated anti-rabbit IgG antibodies (Biolegend) as described previously [91]. All samples were acquired using the BD FACSCalibur or LSR II flow cytometers and analysed by FlowJo software.

### Determination of cytokine release

SFs were seeded at 10^4^ (96-well plates; Corning) and 0.3 x10^6^ (6-well plates) cells per well and after overnight incubation in 1% FCS DMEM to synchronize the cells, stimulated for 24 hours in 10% FCS DMEM with LPS (1 μg/ml, *Salmonella Minnesota*; Sigma-Aldrich), BLP (Pam_3_Cys-Ser-(Lys)_4_; 0.5 μg/ml, Enzo), IL-1β (10 ng/ml, Immunotools) or IL-17 (25 ng/ml, Immunotools) as indicated. Supernatants were collected and analysed using ELISA kits detecting IL-6, CCL-2 and IL-1β (Ready-SET-Go! from eBioscience), with absorbance read at 450nm using a Tecan Sunrise plate reader.

### FACE assays

Fast-activated cell-based (FACE) assays were performed as we described previously [92]. SFs were seeded at 10^4^ cells/well (96-well plates) and after overnight incubation in 1% FCS DMEM to synchronize the cells, were then stimulated for the indicated time points (from 0 to 120 minutes) before being treated with fixative buffer (Biolegend) according to the manufacturer’s recommendations. Once permeabilized, the cells were incubated with antibodies specific for either the activated or whole protein form of the signalling element (e.g., ERK1/2 and the dually phosphorylated active forms of ERK [pERK; Cell Signalling Technology]) for 1 hour before addition of streptavidin-conjugated horseradish peroxidase for 30 minutes (anti-rabbit HRP, Cell Signalling Technology). Following washing and incubation with tetramethylbenzidine (TMB), absorbance was read at 450nm using a Tecan Sunrise plate reader.

### Western Blot analysis

SFs were seeded in 6-well plates at 1x10^6^ cells per well and synchronised overnight in 1% FCS DMEM before being treated as indicated. Following washing, cells were lysed by addition of 100 μl of RIPA buffer (50 nM Tris buffer pH 7.4, 150 mM sodium chloride, 2% (v/v) NP40, 0.25% (w/v) sodium deoxycholate, 1 mM EGTA, 1x Halt protease inhibitor and 1x Halt phosphatase inhibitor [Pierce]) and the protein concentration of the solubilised proteins measured by the BCA protein assay kit (Pierce). Proteins (20 μg) were separated by SDS-PAGE on 4–12% Bis-Tris gradient gels, using the NuPAGE Novex system (Invitrogen) in NuPAGE MOPS buffer, with addition of antioxidant. Proteins were then transferred onto nitrocellulose membranes (GE) using the wet transfer NuPAGE system as described previously [93]. Following blocking (5% non-fat milk protein in Tris buffered saline-1% (v/v) Tween-20 [TBS-T]), membranes were incubated with the relevant primary antibody in 5% BSA TBS-T overnight at 4°C. After washing, secondary antibody conjugated to HRP was added in 5% milk for 1 hour at room temperature and following washing developed using ECL Western blotting substrate (Thermo Fisher Scientific) and X-ray film (Kodak). Membranes were stripped and re-probed using Restore Western Blot stripping buffer (Thermo Fisher Scientific) as described previously [93] and exemplar blots shown (**S7 Fig**).

### mRNA and miRNA analysis

Total RNA and miRNA were isolated and separated using the miRNeasy kit (Qiagen) following the manufacturer’s instructions.

For miRNAs, the miScript Reverse Transcription Kit (Qiagen) was used for cDNA preparation. miScript SYBR green qPCR kit (Qiagen) and miScript primer assay (Qiagen) were used for semi-quantitative determination of the expression of mouse miR-19b-1 (MS00005915), miR19b-1* (MS00024493), miR-19b-2* (MS00024500), miR-23b-2 (MS00032606), miR-24-1* (MS00011543), miR-34a (MS00001428), miR-124*(MS00011081), miR-125a (MS00001533) miR-146 (MS00001638), miR-155 (MS00001701), miR-203 (MS00001848), miR-203* (MS00011452) and miR-346_2 (MS00032753). The expression of Hs-RNU6-2_11 (MS00033740) was used as endogenous control [94]. SFs were transfected with the miR-155 mimic, miR-155-5p or its control scrambled miR (Thermo Scientific Dharmacon) using Effectene (Qiagen) according to the manufacturer’s instructions as described previously [32].

For mRNAs, the high-capacity cDNA reverse transcription kit (Invitrogen) was used and subsequently, qPCRs were performed using TaqmanⓇ gene expression assay kits, with all primers obtained from Life Technologies for the following genes: CCL2 (Mm00441242_m1), DNMT1 (Mm01151063_m1), DNMT3a (Mm00432881_m1), GAPDH (Mm03302249_g1), IL-6 (Mm00446190_m1), MMP9 (Mm00442991_m1) and MMP13 (Mm00439491_m1).

### DNA methylation analysis

Global DNA methylation levels were determined by ELISA-like measurement of methyl cytosine levels. Briefly, genomic DNA was extracted from cells (PureLink Genomic DNA Mini Kit, Invitrogen) and methyl cytosine levels assessed by a specific primary and secondary HRP-conjugated antibody system following the manufacturer’s instructions (Imprint Methylated DNA Quantification Kit-Sigma). Following development with TMB, absorbance was read at 450nm using a Tecan Sunrise plate reader to determine relative global DNA methylation levels. In experiments investigating induced DNA-demethylation of Naïve-SFs following chronic exposure to cytokines, cells were first synchronized overnight in 1% FCS DMEM and then cultured for 14 days in 10% FCS DMEM and stimulated daily with IL-1β (1 ng/ml; Invitrogen) or IL-17 at (2.5 ng/ml; Invitrogen), following the protocol previously published for RASFs [43]. In addition, as a control for the global DNA methylation assay, following synchronisation, SFs were treated daily with the DNMT1 inhibitor, 5-azacytidine (5-aza, Sigma-Aldrich; 1 μM in 10% FCS DMEM) for 7 days according to previous publications [6, 33].

DNA methylome analysis was carried out on SFs (10 x 10^6^ cells/group) from a single CIA model (Naïve; CIA, articular score; 3.17 ± 1.38 and ES-62-CIA, articular score: 0.5 ± 0.22, all groups n = 6), where DNA purification through Illumina sequencing to bioinformatics, was performed by the commercial Active Motif Reduced Representation Bisulfite Sequencing (RRBS) single base pair resolution DNA methylation and bioinformatic service (www.activemotif.com/catalog/1187/rrbs). In order to provide proof of principle of distinct SF phenotypes, analysis was performed on pooled samples from the mice with disease scores most representative of their cohort. This minimises variation inherent to the differences in joint pathology arising in individual mice, a “cohort” strategy we have previously successfully employed to investigate the differential metagenomic signatures of ES-62-treated mice undergoing CIA and obesity-accelerated ageing [10, 87]. Thus, the CIA-SFs were from mice with individual paw articular scores of 3 or 4 whilst those for ES-62-treated CIA- mice were scored 0 or 1.

Briefly, genomic DNA was extracted using the Quick-gDNA MiniPrep kit (Zymo Research D3024) following the manufacturer’s instructions at Active Motif. gDNA (100 ng) was digested with TaqaI (NEB R0149) at 65°C for 2h followed by MspI (NEB R0106) at 37°C overnight. Following enzymatic digestion, libraries were generated using the Ovation RRBS Methyl-Seq System (Tecan 0353–32) following the manufacturer’s instructions. In brief, digested DNA was randomly ligated, and, following fragment end repair, bisulfite converted using the EpiTect Fast DNA Bisulfite Kit (Qiagen 59824). After conversion and clean-up, samples were amplified using the Ovation RRBS Methyl-Seq System protocol for library amplification and purification. Libraries were measured using the Agilent 2200 TapeStation System and quantified using the KAPA Library Quant Kit ABI Prism qPCR Mix (Roche KK4835). Libraries were sequenced on a NextSeq 500 at SE75 and the resulting single-end 75 bp sequencing reads were mapped to the genome using RRBSMAP [95] using default settings (12bp seed length, max number of mismatches = 0.08 x read length), retaining only unique alignments and generating detailed representation of the methylation data (Chromosome; position on sense strand of chromosome containing CpG; Methylation ratio [C_count/eff_CT_count]; C_count; CT_count; rev_G-Count; rev_GA_count and 95% confidence intervals of methylation ratios) at single base resolution, covered by at least 3 unique sequencing reads. Any PCR duplicates based on a randomized 6-mer barcode were removed and alignment information for the remaining reads stored in the resulting BAM files. CpG gene scores in terms of methylation percentages were generated by GBSA 2.0 software [96]. The quality control report of the general sequencing statistics for the samples and analysis of the differentially methylated RRBS DNA fragments (15-1000bp flanked by Mspl and/or Taql restriction enzyme sites) containing at least 2 CpGs are shown (**S8 Fig**). The differential methylation analysis was produced using DMAP software [97] to provide information on gene id, chromosome and fragment parameters (e.g. length, start and end position, number of CpGs) and the regions annotated by Homer software [98] packages respectively, with the Chi-squared test applied. Visualisation of differentially methylated genomic regions was achieved by uploading BED files within the UCSC genome browser. Raw read (FASTq files), GpG gene score and detailed methylation data files (tab-delineated txt files) have been deposited in the NCBI-GEO repository and assigned study accession code GSE158185 (Naïve, GSM4794946; CIA, GSM4794947 and CIA_ES-62, GSM4794948).

For pathway analysis, genes were filtered according to their methylation content, selected to identify “binary” methylation signatures of synovial fibroblasts as follows: (i) naïve ≥ 0.9, CIA ≥ 0.9, ES-62 = 0; (ii) naïve = 0, CIA ≥ 0.9, ES-62 ≥ 0.9; (iii) naïve ≥ 0.9, CIA = 0, ES-62 ≥ 0.9; (iv) naïve ≥0.9, CIA = 0, ES-62 = 0; (v) naïve = 0, CIA ≥ 0.9, ES-62 = 0 and (vi) naïve = 0, CIA = 0, ES-62 ≥ 0.9. Values of 1 represent full methylation of the selected region whilst 0 represents no methylation found. Heatmaps to show these "binary” differential methylation profiles were made using the heatmap function under R environment. In addition, sets of genes (methylation content ≥ 0.9 for each treatment) were uploaded into String software (http://string-db.org, ELIXIR) to identify enriched interactive gene clusters and their associated KEGG pathways and functional gene classifications (summarised by pie-charts).

### Statistics

All statistical analyses were performed using Prism software (GraphPad Software) using the one-tailed Student’s t-test (parametric data) or Mann-Whitney test (non-parametric data) and One- or Two-way ANOVA with appropriate post-test as indicated. Statistical significance is shown as *p<0.05, **p<0.01 and ***p<0.001.

## Supporting information

S1 Fig ES-62 countering of the CIA-SF phenotype can be mimicked by inhibitors of ERK and STAT3 signalling. (DOCX)

S2 Fig ES-62 can directly target SF responses. (DOCX)

S3 Fig Differential CpG methylation of MyD88 and SOCS1. (DOCX)

S4 Fig Analysis of SF methylation phenotypes. (DOCX)

S5 Fig SMAs, 11a and 12b, inhibit pro-inflammatory responses of CIA SFs and 12b mimics the ability of ES-62 to remodel SF responses, *in vitro*. (DOCX)

S6 Fig Representative gating and phenotyping of SFs by flow cytometry. (DOCX)

S7 Fig Exemplar Western Blots. (DOCX)

S8 Fig Sequencing statistics and differential methylation analysis. (DOCX)

S1 Table Differential expression of genes implicated in Rheumatoid Arthritis Synovial Fibroblast pathogenesis. (DOCX)
