# Supplementary material for: Suppression of inflammatory arthritis by the parasitic worm product ES-62 is associated with epigenetic changes in synovial fibroblasts
Source: PLoS Pathog. 2021 Nov 8;17(11):e1010069. doi: 10.1371/journal.ppat.1010069 (PMC8601611; doi:10.1371/journal.ppat.1010069)
Supplement: S1 Fig — (DOCX) [file ppat.1010069.s001.docx]

**S1 Fig. ES-62 countering of the CIA-SF phenotype can be mimicked by inhibitors of ERK and STAT3 signalling.** (**A and B**) Spontaneous (None), IL-17- and LPS-stimulated CCL2 responses were elevated in CIA-, relative to Naïve-SFs and reduced in ES-62-CIA (ES-62)-SFs. Data are from single experiments representative of three. SFs were analysed for mRNA levels of (**C**) IL-6 and (**D**) CCL2 where data are from single experiments representative of at least two and presented as mean (of means of triplicates) values ± SEM of n=3 independent cultures, assayed by qRT-PCR. The CIA controls for the IL-6 and CCL2 mRNA data were the same as those used in **Fig 8**. Throughout, SFs were pooled from individual mice to generate representative explant cohorts with articular scores: (**A**) CIA, 8.5 ± 1.19, n=4; ES-62, 0.8 ± 0.49, n=5; (**B**) CIA, 3.17 ± 1.38, n=6; ES-62, 0.5 ± 0.22, n=6; (**C and D)** CIA, 3.66 ± 1.5, n=6; ES-62, 1.5 ± 1.15, n=6. In all panels, *p<0.05; **p<0.01; ***p<0.001 relative to CIA-SFs. (**E**) Preincubation with the MEK inhibitor, PD98059 (iERK; 25 µM) for 2 h blocked IL-17-stimulated ERK activation in Naïve-SFs as determined by analysis of the pERK/ERK ratio measured by the FACE assay and where data represent mean values ± SEM of n=3 independent cultures and **p<0.01 and ***p<0.001 are relative to the IL-17-stimulated control (“IL-17-None”) sample. **(F)** Naïve SFs were pre-incubated with medium containing the STAT3 inhibitor 5.15 DPP (iSTAT3; 50 µM, +) or medium alone (-) for two hours prior to stimulation for 20 min with IL-17 and STAT3 and ERK activation assessed by Western blot analysis of pSTAT3, pERK and GAPDH expression, with each lane representing independent cultures. Expression was quantitated relative to GAPDH and showed that treatment with iSTAT3 resulted in an average inhibition of 44% of STAT3, but only 7% of ERK, activity relative to the control cells. (**G**) Naïve-SFs were pretreated with iERK or iSTAT3 for two hours priors to incubation in medium alone (None) or with IL-17 overnight and levels of MMP13 mRNA measured. Data are shown as mean values ± SEM, n=3 independent cultures and where *p<0.05 is relative to the “IL-17-None” control. (**H**) SFs from Naïve, CIA- (articular score; 2.8 ± 1, n=7) and ES-62-CIA (articular score; 0.8 ± 0.8, n=5) mice were stimulated with IL-17 for the indicated times and ERK activation (pERK/ERK ratio), determined by FACE assay: data are means ± SEM, n=3 independent cultures and are representative of two independent experiments, *p<0.05 and **p<0.01 for CIA- versus Naïve-SFs. (**I**) SFs from Naïve- (N), CIA- (C) and ES-62-CIA (E) mice were analysed for expression of pSTAT3 following stimulation with IL-17 for the indicated times (min): each lane represents an individual culture with the relative pSTAT3 expression (pSTAT3/GAPDH ratio normalized to the Naïve (N) zero-time control) shown below the GAPDH panel.

**
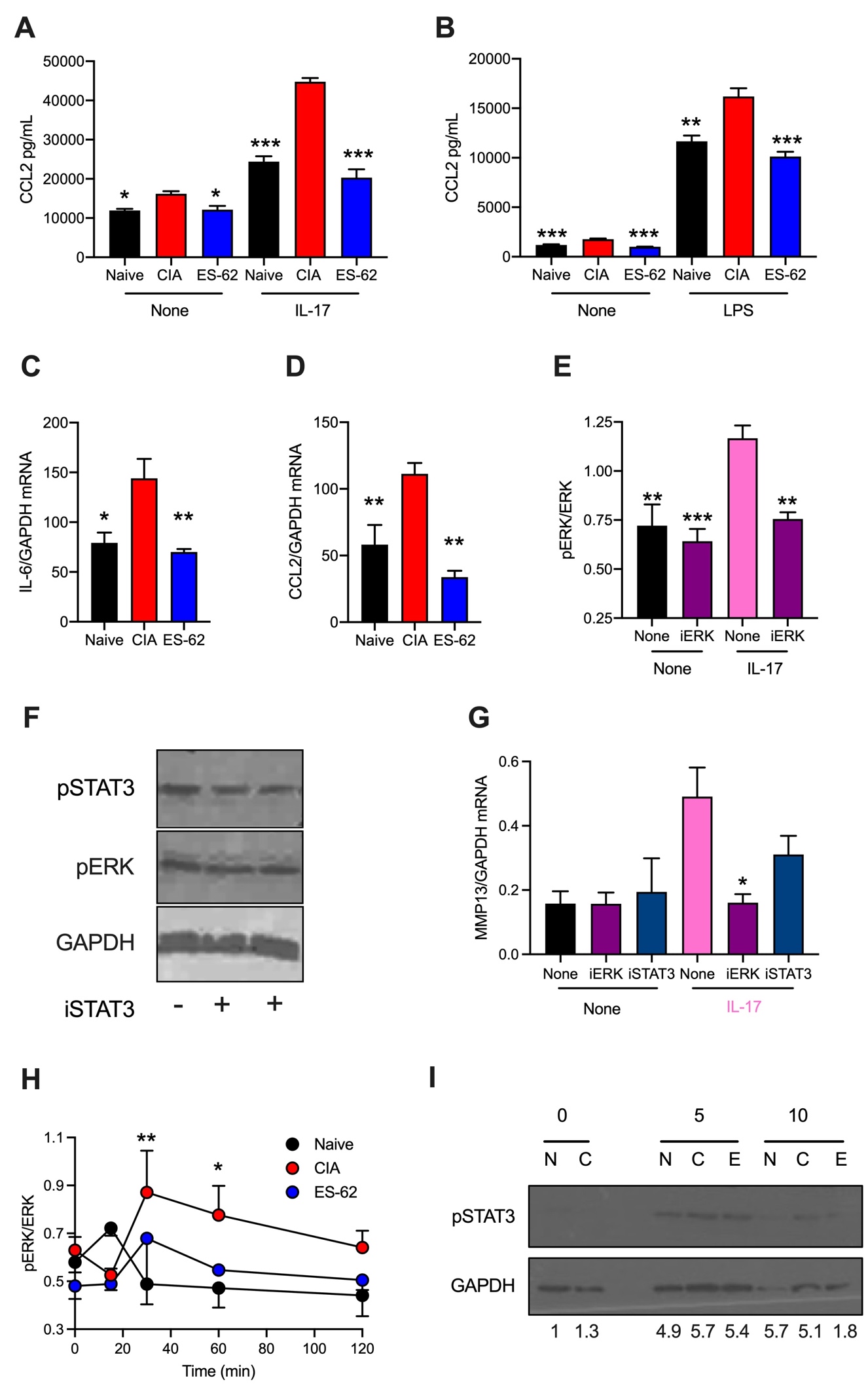
**
