# Supplementary material for: Suppression of inflammatory arthritis by the parasitic worm product ES-62 is associated with epigenetic changes in synovial fibroblasts
Source: PLoS Pathog. 2021 Nov 8;17(11):e1010069. doi: 10.1371/journal.ppat.1010069 (PMC8601611; doi:10.1371/journal.ppat.1010069)

**S2 Fig. ES-62 can directly target SF responses.** (**A**) Following preliminary *in vitro* dose response studies, Naïve and CIA (articular score, 2.86 ± 1.01, n=7) SFs were pre-treated with PBS or ES-62 (1 µg/ml) for 2 h prior to stimulation with PBS or IL-17 (Naïve SFs only; IL-17) for 24 h and then IL-6 release measured by ELISA. Data are presented as the mean values ± SEM of 2-4 independent cultures, each of which were assayed in triplicate and where **p<0.01 compares CIA- versus +ES-62 CIA-SFs and ***p<0.001 compares IL-17-stimulated Naïve-SFs treated with PBS versus those exposed to ES-62. (**B and C**) Naïve SFs were pre-treated with PBS or ES-62 (1 µg/ml) for 24 h prior to stimulation with PBS (None) or IL-17 for 20 min and then (**B**) ERK (pERK/ERK ratio) and (**C**) STAT3 (pSTAT3/STAT3 ratio) activation determined by FACE assay. Data are expressed as means ± SEM, n=3 independent cultures where *p<0.05 for IL-17+ES-62 versus IL-17 SFs (**B**) and **p<0.01 for IL-17 SFs versus all other groups (**C**). Naïve (**D-F**) and CIA (**G-H**; 2.86 ± 1.01, n=7) SFs were pre-treated with PBS or ES-62 (1 µg/ml) for 24 h prior to stimulation with PBS (None) or IL-17 (IL-17) for 20 min and then ERK activation (pERK/ERK ratio) determined by flow cytometry. (**J-L**) IL-1R1, TLR4 and MyD88 mRNA levels were determined in explant SF cultures from Naïve, CIA and ES-62-CIA mice. SFs were pooled from individual mice to generate representative explant cohorts with articular scores: (**J**) CIA, 8.5 ± 1.19, n=4 and ES-62-CIA, 0.8 ± 0.49, n=5 (**K and L**) CIA, 4.75 ± 1.31, n=8 and ES-62-CIA 0, n=6. (**M and N**) MMP9 and MMP13 mRNA levels in explant SFs (articular scores, CIA: 3.66 ± 1.5, n=6; CIA + IL-1β: 6 ± 1.93, n=6; the CIA controls for the MMP9 and MMP13 mRNA data were the same as those used in **Fig 8)** are presented as mean values ± SEM for n=3 independent cultures and **p<0.05 and ***p<0.001 are relative to the CIA group.


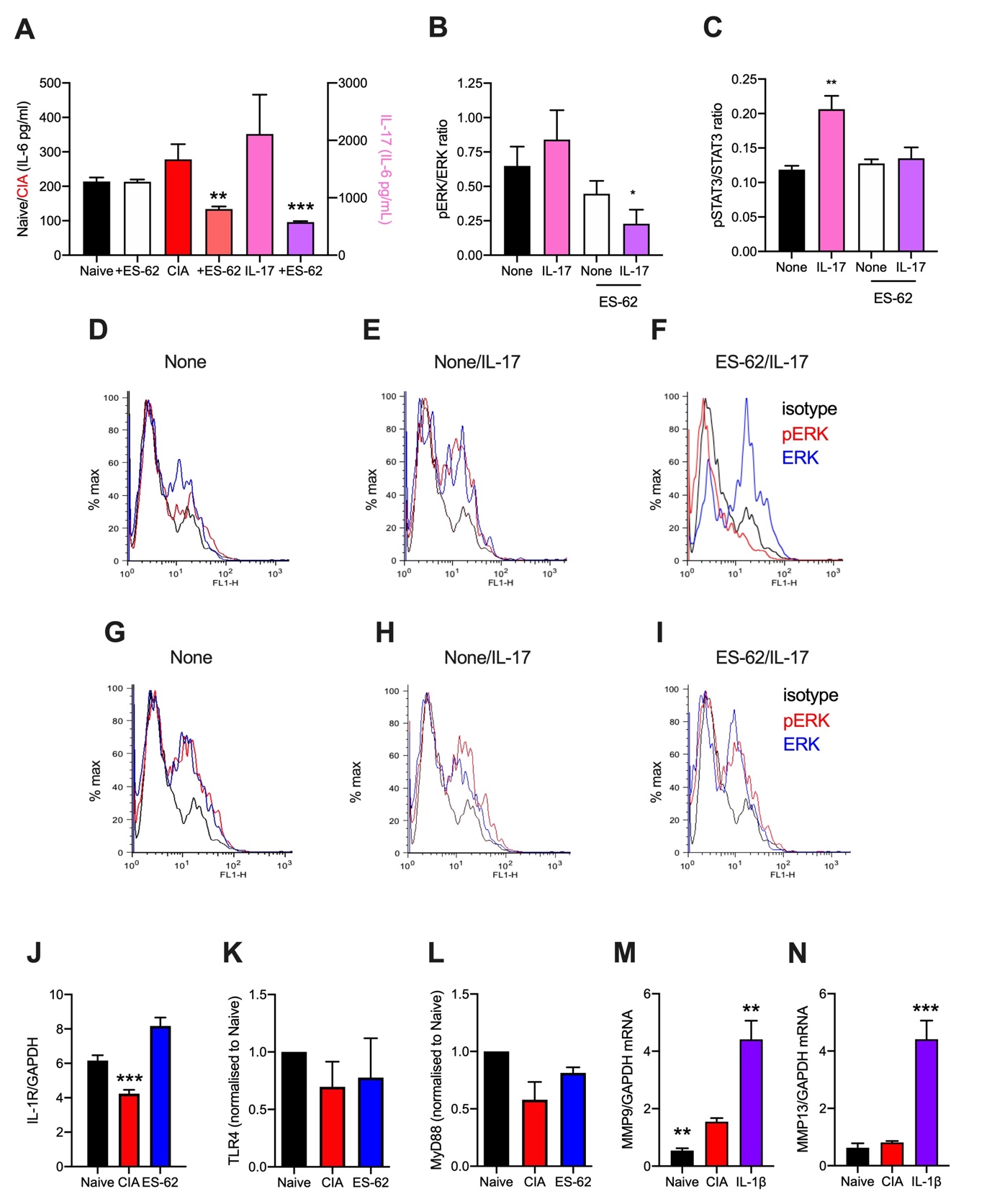

Supplement: S2 Fig — (DOCX) [file ppat.1010069.s002.docx]
