# Supplementary material for: Suppression of inflammatory arthritis by the parasitic worm product ES-62 is associated with epigenetic changes in synovial fibroblasts
Source: PLoS Pathog. 2021 Nov 8;17(11):e1010069. doi: 10.1371/journal.ppat.1010069 (PMC8601611; doi:10.1371/journal.ppat.1010069)

**S3 Fig.** **Differential CpG methylation of MyD88 and SOCS1.** Visualisation of the exon 3 region of MyD88 (**A**) and Promoter/TSS regions of SOCS1 (**B**) showing their differential CpG methylation in Naïve, CIA and ES-62-CIA cohorts with images created by uploading BED files in the UCSC browser.


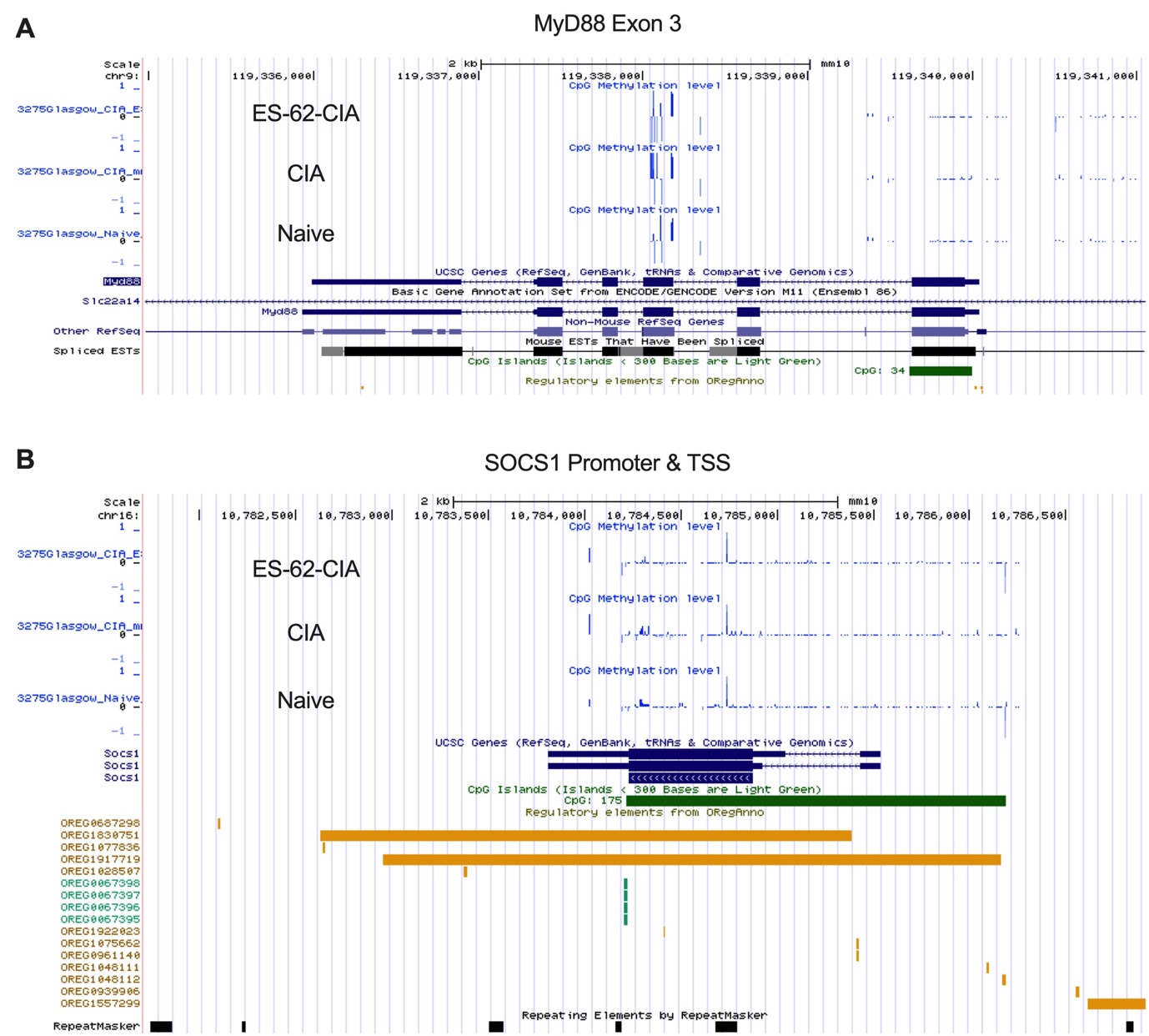

Supplement: S3 Fig — (DOCX) [file ppat.1010069.s003.docx]
