# Supplementary material for: Suppression of inflammatory arthritis by the parasitic worm product ES-62 is associated with epigenetic changes in synovial fibroblasts
Source: PLoS Pathog. 2021 Nov 8;17(11):e1010069. doi: 10.1371/journal.ppat.1010069 (PMC8601611; doi:10.1371/journal.ppat.1010069)

**S4 Fig. Analysis of SF DNA methylation phenotypes.** Genes displaying >0.9 methylation of CpG sites in the Promoter3K (**A**) and Gene Body (**B**) regions were modelled by String pathway analysis software: the differential pathway interactions predicted by over-representational analysis for the Naïve, CIA and ES-62-CIA (ES-62) cohorts and the functional classification of the key cluster genes highlighted (summarized by pie-chart analysis according to the accompanying colour code), are also shown. Predicted differential KEGG pathway interactions, ranked on false discovery rate (fdr) q-values are shown for both the Promoter3K and Gene Body regions (**C**).

String pathway analysis of the fully methylated genes predicted quite distinct interactions of functionally disparate classes of genes. For example, whilst the “silenced” (hypermethylated) genes associated with Promoter3K of the Naïve-SF group were predominantly epigenetic elements (chromatin and histone modifiers) and ubiquitin (regulation of protein stability and trafficking) pathway components, reflecting their rewired phenotype many of these elements were relatively hypomethylated in SFs from the CIA group. Instead, CIA-SFs displayed DNA hypermethylation of a wider spectrum of gene classes, particularly those associated with (dys)regulation of inflammation, proliferation and cell migration, as well as metabolism and REDOX homeostasis. Strikingly, exposure to ES-62 resulted in SFs exhibiting a fully DNA methylated promoter profile quite distinct to that of either the Naïve or CIA SFs: here Promoter3K DNA hypermethylation predominantly reflected silencing of genes functionally associated with cilia, sensory organelles which play roles in coordinating development and tissue repair/regeneration and cell migration, differentiation, autophagy and signal transduction responses. Such pathway analysis likewise highlighted that exposure to ES-62 *in vivo* resulted in SFs displaying stable profiles of Gene Body DNA hypermethylation quite distinct to those of both Naïve- and CIA-SFs. KEGG analysis, which highlighted genes associated with control of pathways dysregulated in Rheumatoid Arthritis being fully methylated only in Naïve SFs, clearly illustrated the cohort-specific enrichment of pathways associated with the fully methylated Promoter3K and Gene Body regions. Indeed, there was minimal commonality observed amongst the groups, suggesting this was limited essentially to differential epigenetic regulation of positive and negative regulatory elements of pathways regulating chronic inflammatory disease states (alcoholism, inflammatory bowel disease and systemic lupus erythematosus [SLE]) and their associated inflammatory cytokine and chemokine networks.


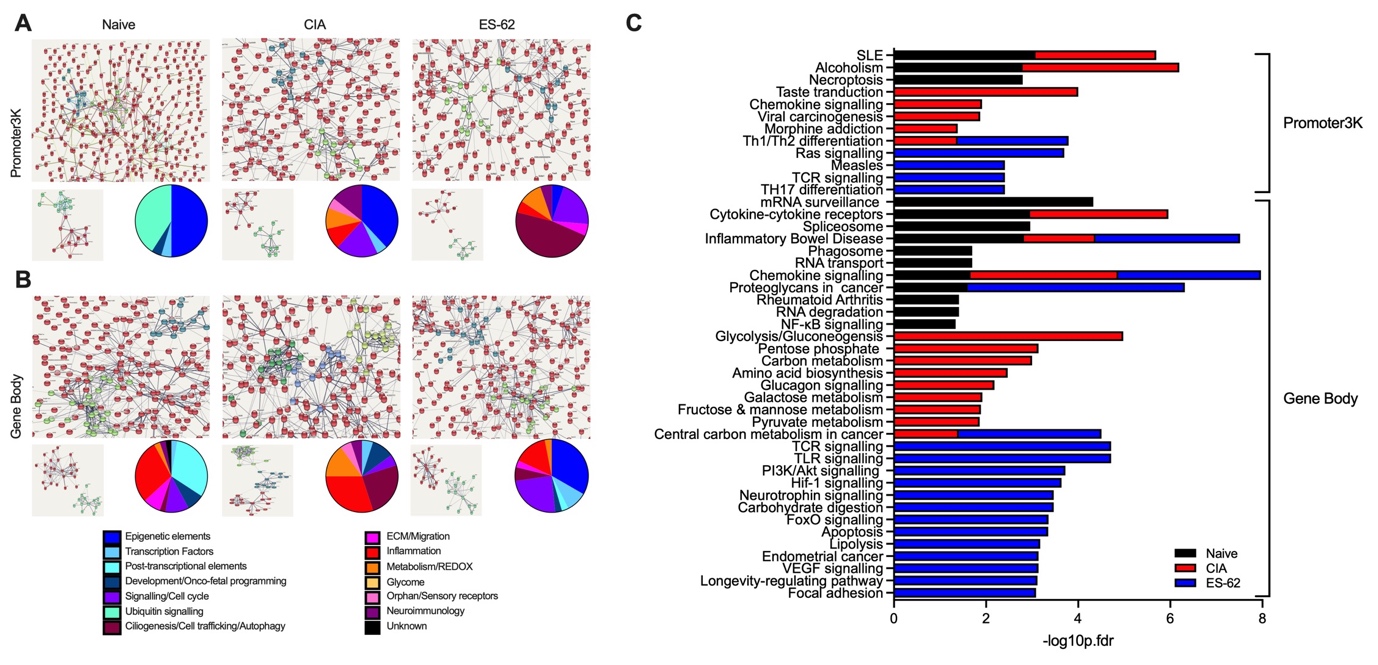

Supplement: S4 Fig — (DOCX) [file ppat.1010069.s004.docx]
