# Supplementary material for: Suppression of inflammatory arthritis by the parasitic worm product ES-62 is associated with epigenetic changes in synovial fibroblasts
Source: PLoS Pathog. 2021 Nov 8;17(11):e1010069. doi: 10.1371/journal.ppat.1010069 (PMC8601611; doi:10.1371/journal.ppat.1010069)

**S5 Fig.** **SMAs, 11a and 12b, inhibit pro-inflammatory responses of CIA-SFs and** **12b mimics the ability of ES-62 to remodel SF responses, *in vitro*.** CIA-SFs were pretreated with medium (None) or the SMAs (5 µg/ml) overnight prior to incubation of the Naïve and CIA groups in medium or medium + LPS (1 µg/ml) as indicated and levels of IL-6 (**A**) or CCL2 (**B**) in culture supernatants determined after 24 h or those of miR-155 (**C**) after 6h. Data are presented as the mean ± SEM values of at least 3 independent cultures. (**D and E**) Naïve-SFs were incubated in medium or medium + IL-1β (1 ng/ml) in the presence or absence of 12b (100 ng/ml), refreshing the medium/stimuli each day, for 14 days. The cohorts of SFs were then assayed for their global DNA methylation status (**D**) and associated spontaneous release of IL-6 (**E**). Data are presented as mean ± range values for two independent cultures (except for the None/IL-1β global DNA methylation data (**D**) that were from a single culture). For statistical analysis, *p<0.05, **p<0.01 and ***p<0.001 are for the indicated responses relative to the CIA group.


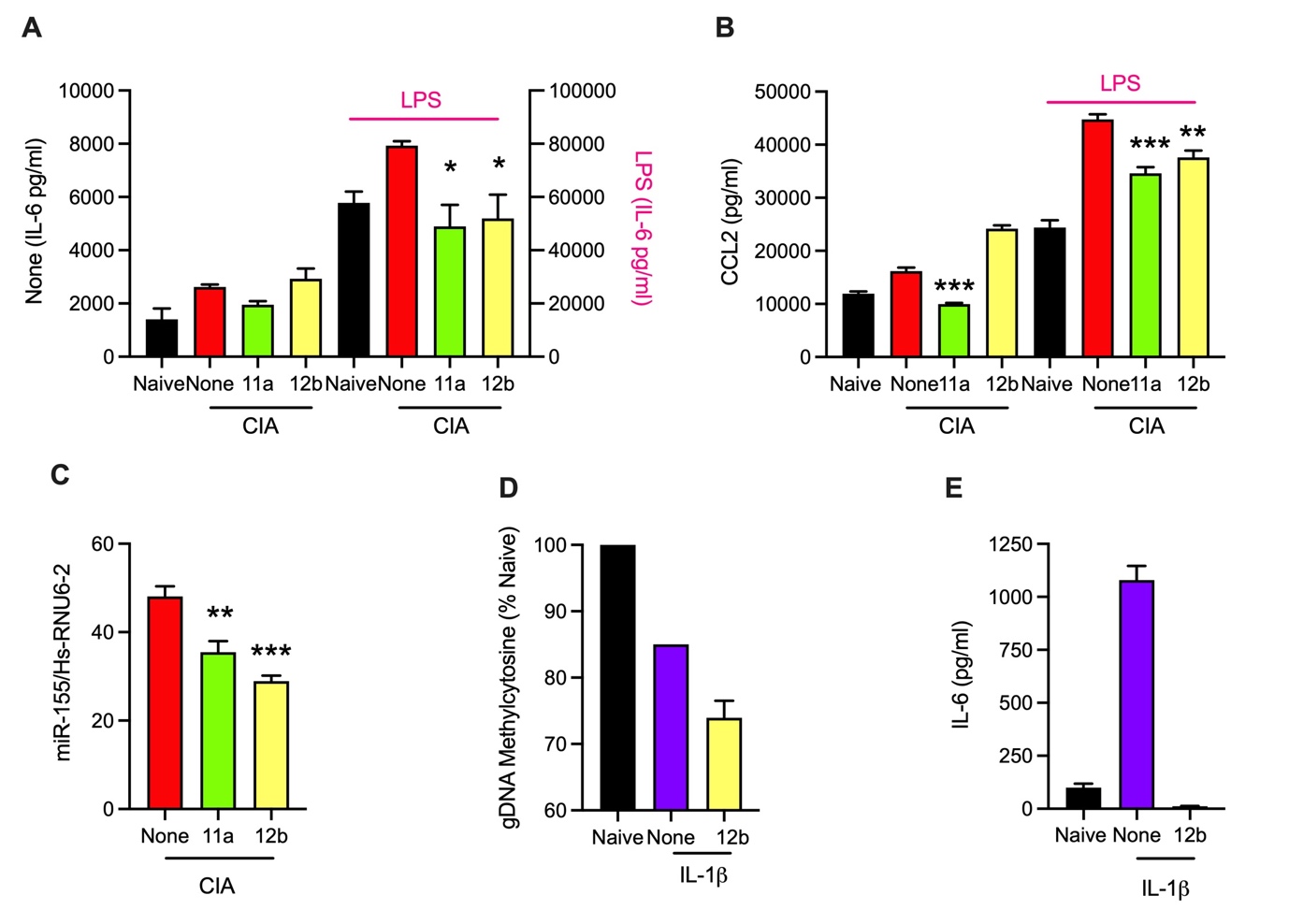

Supplement: S5 Fig — (DOCX) [file ppat.1010069.s005.docx]
