# Supplementary material for: Suppression of inflammatory arthritis by the parasitic worm product ES-62 is associated with epigenetic changes in synovial fibroblasts
Source: PLoS Pathog. 2021 Nov 8;17(11):e1010069. doi: 10.1371/journal.ppat.1010069 (PMC8601611; doi:10.1371/journal.ppat.1010069)

**S6 Fig. Representative gating and phenotyping of SFs by flow cytometry.** SFs were isolated according to the protocol of Armaka *et al* 2009 as described previously in the Materials and Methods section and then expanded in explant cultures for 3-4 weeks. Following gating of live cells on the basis of their FSC-A and SSC-A profile, SF were confirmed as being positive for CD90.2 (Thy1.2), CD106 (VCAM-1) and CD54 (ICAM-1) by flow cytometry using antibodies specific to these markers (black lines) relative to relevant isotype control antibodies (grey lines).


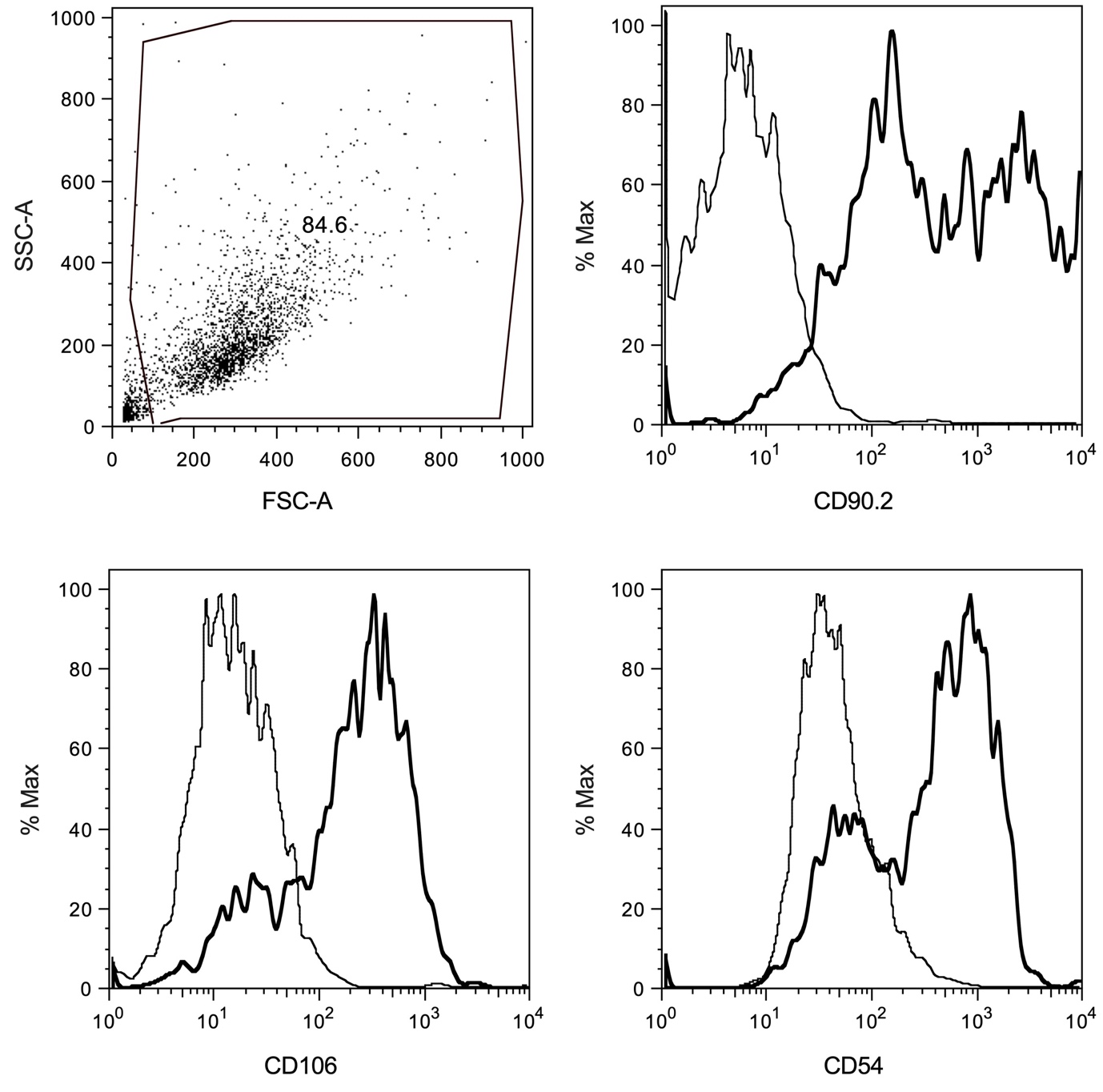

Supplement: S6 Fig — (DOCX) [file ppat.1010069.s006.docx]
