# Supplementary material for: Suppression of inflammatory arthritis by the parasitic worm product ES-62 is associated with epigenetic changes in synovial fibroblasts
Source: PLoS Pathog. 2021 Nov 8;17(11):e1010069. doi: 10.1371/journal.ppat.1010069 (PMC8601611; doi:10.1371/journal.ppat.1010069)

**S7 Fig. Exemplar Western Blots.** Representative uncropped images of the Western Blot analysis of expression of DNMT1 (**A and C**), GAPDH (**B, C, and E**), ERK-1& -2 (**C**), pSTAT3 (**D**) and pERK-1 & -2 (**F**) are shown with molecular marker standards. In (**B**) the blot has been stained for DNMT1, HDAC1 and GAPDH and in (**C**) the membrane was cut at ~60 kDa to simultaneously stain for DNMT1 (top image) and loading control ERK-1 and -2 (middle image). The lower molecular weight portion was also subsequently stripped and reprobed for GAPDH and resultant cropped film visualised (lower image). The data shown represent the effects of chronic cytokine stimulation on DNMT1 expression (**Fig 2E**; panels A & B) and pSTAT3 and pERK activation (**Fig 3C**, panels D-F). The conditions presented in these figures (Naïve, IL-1β and IL-17) have the relevant lanes highlighted in the full-length blots. In addition, the differential expression of DNMT1 in Naïve- and CIA- (and ES-62-CIA-) SFs presented in **Fig 2D** is derived from the blots shown here in panel C.


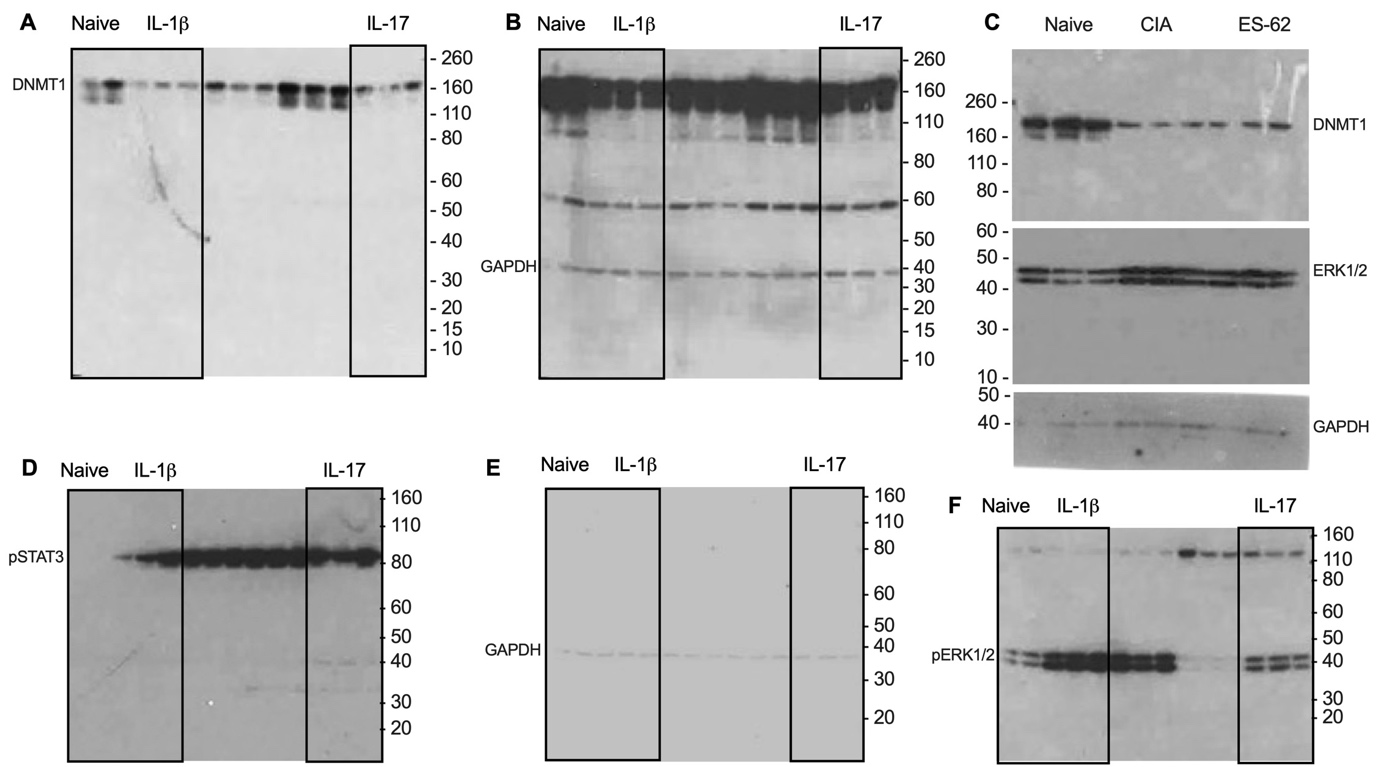

Supplement: S7 Fig — (DOCX) [file ppat.1010069.s007.docx]
