# Supplementary material for: Suppression of inflammatory arthritis by the parasitic worm product ES-62 is associated with epigenetic changes in synovial fibroblasts
Source: PLoS Pathog. 2021 Nov 8;17(11):e1010069. doi: 10.1371/journal.ppat.1010069 (PMC8601611; doi:10.1371/journal.ppat.1010069)

**S8 Fig. Sequencing Statistics and differential methylation analysis.** Quality control report of (**A**) general sequencing statistics and the (**B**) differential methylation analysis generated using DMAP software for the samples within the study. A total of 25132 differentially methylated RRBS fragments containing at least 2CpGs were mapped with the proportion of their locations identified using Homer software annotation and where “Promoter” is defined by -1000bp to +100bp relative to RefSeq Transcription Start Site (TSS). The global coverage of sequencing depth for individual CpGs on sense and antisense strands (**C**), the numbers of genes for which regions contain ≥3 CpGs covered by ≥3 sequencing reads (**D**) and the percentage of CpG sequenced in these regions within loci (**E**) are shown for each of the Naïve-, CIA- and ES-62-CIA-SF cohorts. The regions are defined as Promoter3K (0-3000bp upstream of TSS); Promoter2K (0-2000bp upstream of TSS; TSS (-1000bp to +1000bp of TSS); 1^st^Exon-Intron (start of 1^st^ exon to end of 1^st^ intron of gene) and Gene Body (start of 5’UTR to end of 3’UTR) and images (**C-E**) were generated by the GBSA 2.0 software package.


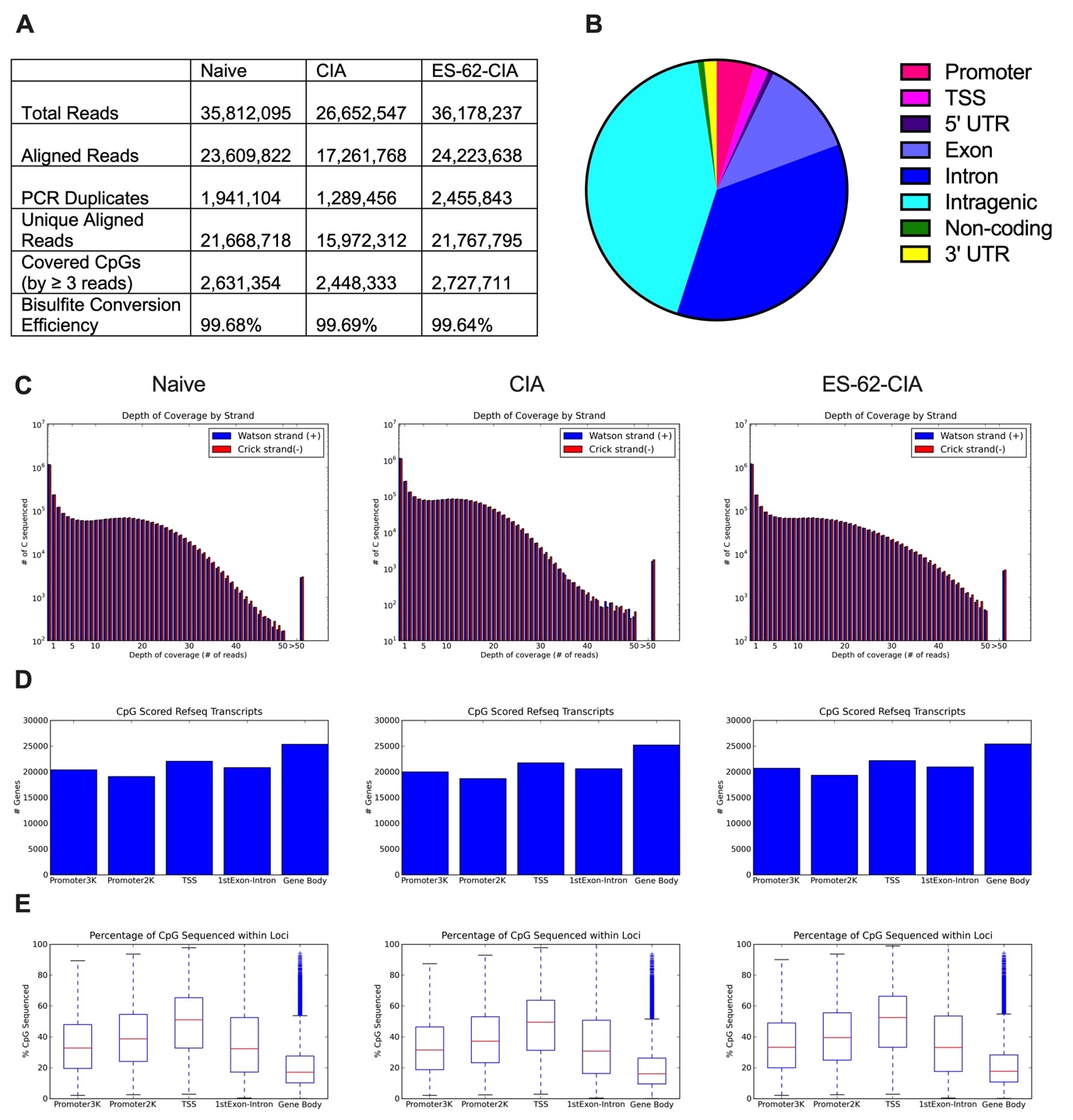

Supplement: S8 Fig — (DOCX) [file ppat.1010069.s008.docx]
