# Supplementary material for: Suppression of inflammatory arthritis by the parasitic worm product ES-62 is associated with epigenetic changes in synovial fibroblasts
Source: PLoS Pathog. 2021 Nov 8;17(11):e1010069. doi: 10.1371/journal.ppat.1010069 (PMC8601611; doi:10.1371/journal.ppat.1010069)
Supplement: S1 Table — (DOCX) [file ppat.1010069.s009.docx]

**S1 Table: Differential expression of genes implicated in Rheumatoid Arthritis Synovial Fibroblast pathogenesis.** The classes of genes are colour-coded according to the pie-charts in Figs 7 and 8, whilst the treatment groups are coloured red and blue to reflect the differential methylation at the particular sites, as highlighted in the ***heatmaps*** (Fig 8). In addition, genes in clusters identified by ***pathway analysis*** of regions exhibiting >90% methylation are coloured red whilst white indicates 0< methylation <90% in the relevant treatment groups.

| **Gene id** | **Site** | **Naive** | **CIA** | **ES-62** | **Function** |
| --- | --- | --- | --- | --- | --- |
|  |  |  |  |  | **miRs** |
| *miR134* | Promoter |  |  |  | miR134 contributes to signature miR panel discriminating RA from SLE and healthy individuals [1]. |
| *miR146* | Promoter |  |  |  | miR important to SF pathogenesis [2-5] |
|  |  |  |  |  | **Transcription Factors** |
| *Batf* | Gene Body |  |  |  | Upregulated in inflammatory arthritis - KO ablates pannus formation [6] |
| *Runx3* | Promoter |  |  |  | SNP/epigenetic (methylation) disruption of multiple Runx3 sites in RA [7] |
|  |  |  |  |  | **Epigenetic Elements** |
| *Piwil2* | Promoter |  |  |  | Piwi-like RNA-mediated gene silencing induced by IL-1β and TNFα [8] |
|  |  |  |  |  | **Signalling** |
| *Jak1* | Promoter |  |  |  | Tyr kinase expressed in SFs and approved drug target (Upadacitinib) in RA [9] |
| *Lair1* | Gene body |  |  |  | Leucocyte-associated immunoglobulin-like receptor-1 inhibits SF invasion and pathogenic responses. Soluble form elevated in RA synovia [10] |
| *Pak1* | Promoter |  |  |  | Ser/Thr kinase implicated in SF migration/invasion in RA [11] |
| *Pik3ca* | Promoter |  |  |  | PI3K, p110α (suppressed by miR124a) promotes RA [12] |
|  |  |  |  |  | **Ubiquitin signalling** |
| *Cul4b* | Promoter |  |  |  | Ubiquitin Ligase that promotes adjuvant arthritis via WNT signalling in SFs [13] |
|  |  |  |  |  | **Autophagy** |
| *Map1lc3b* | Promoter |  |  |  | LC3 - promotes pathogenic RASF responses [14, 15], CIA in rats [16] and citrullination [17] |
|  |  |  |  |  | **ECM/Migration** |
| *Aspn* | Gene Body |  |  |  | Asporin (ECM protein) - susceptibility gene for RA implicated in EMT [18, 19] |
| *TNXb* | Promoter |  |  |  | Tenascin - ECM component, implicated in RA by GWAS and differentially methylated in RA v OA patients [20] |
|  |  |  |  |  | **Inflammation** |
| *Ccl25* | Gene body |  |  |  | Stimulates pathogenic SF responses and receptor (CCR9) KO blocks CIA [21] |
| *Ccl28* | Gene body |  |  |  | Contributes to angiogenesis in RA [22] |
| *Cx3cr1* | Gene body |  |  |  | Fractalkine receptor expressed by SFs; promotes arthritis [23] |
| *Il18* | Gene body |  |  |  | Increased in RA SFs & IL-18R KO suppresses CIA [24] |
| *MMP12* | Promoter |  |  |  | Promotes SF pathogenesis/joint destruction [25] |
| *Spag16* | Promoter |  |  |  | Variants implicated in regulation of MMP3 and joint destruction in RA [26] |
| *Tlr4* | Gene body |  |  |  | Perpetuates synovial inflammation [27] |
| *Tlr7* | Gene body |  |  |  | TLR7: Role in SF-driven osteoclastogenesis [27, 28] |
|  |  |  |  |  | **Metabolism** |
| *Adipoq* | Gene body |  |  |  | Adiponectin, elevated in RA serum and promotes IL-6 production by SFs [29] |
| *Pck1* | Gene Body |  |  |  | Phosphoenolpyruvate carboxy kinase 1 (promotes gluconeogenesis): downregulated in CIA [30] |
|  |  |  |  |  | **Neuroimmunology** |
| *Cort* | Promoter |  |  |  | Cortistatin – acts on SFs to suppress CIA [31] |

**Cited References**

1. Ormseth MJ, Solus JF, Sheng Q, Ye F, Wu Q, Guo Y, et al. Development and Validation of a MicroRNA Panel to Differentiate Between Patients with Rheumatoid Arthritis or Systemic Lupus Erythematosus and Controls. The Journal of Rheumatology. 2020;47(2):188-96.

2. Evangelatos G, Fragoulis GE, Koulouri V, Lambrou GI. MicroRNAs in rheumatoid arthritis: From pathogenesis to clinical impact. Autoimmunity Reviews. 2019;18(11):102391.

3. Hong W, Zhang P, Wang X, Tu J, Wei W. The Effects of MicroRNAs on Key Signalling Pathways and Epigenetic Modification in Fibroblast-Like Synoviocytes of Rheumatoid Arthritis. Mediators Inflamm. 2018;2018:9013124.

4. Sharma AR, Sharma G, Lee SS, Chakraborty C. miRNA-Regulated Key Components of Cytokine Signaling Pathways and Inflammation in Rheumatoid Arthritis. Med Res Rev. 2016;36(3):425-39.

5. Su LC, Huang AF, Jia H, Liu Y, Xu WD. Role of microRNA-155 in rheumatoid arthritis. International Journal of Rheumatic Diseases. 2017;20(11):1631-7.

6. Park SH, Rhee J, Kim SK, Kang JA, Kwak JS, Son YO, et al. BATF regulates collagen-induced arthritis by regulating T helper cell differentiation. Arthritis Research & Therapy. 2018;20(1):161.

7. Webster AP, Plant D, Ecker S, Zufferey F, Bell JT, Feber A, et al. Increased DNA methylation variability in rheumatoid arthritis-discordant monozygotic twins. Genome Med. 2018;10(1):64.

8. Plestilova L, Neidhart M, Russo G, Frank-Bertoncelj M, Ospelt C, Ciurea A, et al. Expression and Regulation of PIWIL-Proteins and PIWI-Interacting RNAs in Rheumatoid Arthritis. PLoS ONE. 2016;11(11):e0166920.

9. Tanaka Y. A review of upadacitinib in rheumatoid arthritis. Modern rheumatology / the Japan Rheumatism Association. 2020;30(5):779-87.

10. Zhang Y, Wang S, Dong H, Yi X, Zhang J, Liu X, et al. LAIR-1 shedding from human fibroblast-like synoviocytes in rheumatoid arthritis following TNF-alpha stimulation. Clinical and Experimental Immunology. 2018;192(2):193-205.

11. Fu D, Yang Y, Xiao Y, Lin H, Ye Y, Zhan Z, et al. Role of p21-activated kinase 1 in regulating the migration and invasion of fibroblast-like synoviocytes from rheumatoid arthritis patients. Rheumatology (Oxford). 2012;51(7):1170-80.

12. Yang B, Ge Y, Zhou Y, Wang J, Xie X, Li S, et al. miR-124a inhibits the proliferation and inflammation in rheumatoid arthritis fibroblast-like synoviocytes via targeting PIK3/NF-kappaB pathway. Cell Biochem Funct. 2019;37(4):208-15.

13. Miao C, Chang J, Zhang G, Yu H, Zhou L, Zhou G, et al. CUL4B promotes the pathology of adjuvant-induced arthritis in rats through the canonical Wnt signaling. J Mol Med (Berl). 2018;96(6):495-511.

14. He SD, Huang SG, Zhu HJ, Luo XG, Liao KH, Zhang JY, et al. Oridonin suppresses autophagy and survival in rheumatoid arthritis fibroblast-like synoviocytes. Pharm Biol. 2020;58(1):146-51.

15. Dinesh P, Rasool M. Berberine mitigates IL-21/IL-21R mediated autophagic influx in fibroblast-like synoviocytes and regulates Th17/Treg imbalance in rheumatoid arthritis. Apoptosis. 2019;24(7-8):644-61.

16. Deng H, Zheng M, Hu Z, Zeng X, Kuang N, Fu Y. Effects of daphnetin on the autophagy signaling pathway of fibroblast-like synoviocytes in rats with collagen-induced arthritis (CIA) induced by TNF-alpha. Cytokine. 2020;127:154952.

17. Sugawara E, Kato M, Kudo Y, Lee W, Hisada R, Fujieda Y, et al. Autophagy promotes citrullination of VIM (vimentin) and its interaction with major histocompatibility complex class II in synovial fibroblasts. Autophagy. 2020;16(5):946-55.

18. Ikegawa S. Expression, regulation and function of asporin, a susceptibility gene in common bone and joint diseases. Current Medicinal Chemistry. 2008;15(7):724-8.

19. Torres B, Orozco G, Garcia-Lozano JR, Oliver J, Fernandez O, Gonzalez-Gay MA, et al. Asporin repeat polymorphism in rheumatoid arthritis. Ann Rheum Dis. 2007;66(1):118-20.

20. Anaparti V, Agarwal P, Smolik I, Mookherjee N, Elgabalawy H. Whole Blood Targeted Bisulfite Sequencing Validates Differential Methylation in C6ORF10 gene of Patients with Rheumatoid Arthritis. The Journal of Rheumatology. 2019.

21. Yokoyama W, Kohsaka H, Kaneko K, Walters M, Takayasu A, Fukuda S, et al. Abrogation of CC chemokine receptor 9 ameliorates collagen-induced arthritis of mice. Arthritis Research & Therapy. 2014;16(5):445.

22. Chen Z, Kim SJ, Essani AB, Volin MV, Vila OM, Swedler W, et al. Characterising the expression and function of CCL28 and its corresponding receptor, CCR10, in RA pathogenesis. Ann Rheum Dis. 2015;74(10):1898-906.

23. Sawai H, Park YW, He X, Goronzy JJ, Weyand CM. Fractalkine mediates T cell-dependent proliferation of synovial fibroblasts in rheumatoid arthritis. Arthritis and Rheumatism. 2007;56(10):3215-25.

24. Marotte H, Tsou PS, Rabquer BJ, Pinney AJ, Fedorova T, Lalwani N, et al. Blocking of interferon regulatory factor 1 reduces tumor necrosis factor alpha-induced interleukin-18 bioactivity in rheumatoid arthritis synovial fibroblasts by induction of interleukin-18 binding protein a: role of the nuclear interferon regulatory factor 1-NF-kappaB-c-jun complex. Arthritis and Rheumatism. 2011;63(11):3253-62.

25. Ye S, Patodi N, Walker-Bone K, Reading I, Cooper C, Dennison E. Variation in the matrix metalloproteinase-3, -7, -12 and -13 genes is associated with functional status in rheumatoid arthritis. Int J Immunogenet. 2007;34(2):81-5.

26. Knevel R, Klein K, Somers K, Ospelt C, Houwing-Duistermaat JJ, van Nies JA, et al. Identification of a genetic variant for joint damage progression in autoantibody-positive rheumatoid arthritis. Ann Rheum Dis. 2014;73(11):2038-46.

27. Elshabrawy HA, Essani AE, Szekanecz Z, Fox DA, Shahrara S. TLRs, future potential therapeutic targets for RA. Autoimmunity reviews. 2017;16(2):103-13.

28. Kim KW, Kim BM, Won JY, Lee KA, Kim HR, Lee SH. Toll-like receptor 7 regulates osteoclastogenesis in rheumatoid arthritis. J Biochem. 2019;166(3):259-70.

29. Liu R, Zhao P, Zhang Q, Che N, Xu L, Qian J, et al. Adiponectin promotes fibroblast-like synoviocytes producing IL-6 to enhance T follicular helper cells response in rheumatoid arthritis. Clinical and Experimental Rheumatology. 2020;38(1):11-8.

30. Zhao Y, Yan X, Li X, Zheng Y, Li S, Chang X. PGK1, a glucose metabolism enzyme, may play an important role in rheumatoid arthritis. Inflamm Res. 2016;65(10):815-25.

31. Gonzalez-Rey E, Chorny A, Del Moral RG, Varela N, Delgado M. Therapeutic effect of cortistatin on experimental arthritis by downregulating inflammatory and Th1 responses. Ann Rheum Dis. 2007;66(5):582-8.
